# Selection of Lentil (*Lens Culinaris* (Medik.)) Genotypes Suitable for High-Temperature Conditions Based on Stress Tolerance Indices and Principal Component Analysis

**DOI:** 10.3390/life12111719

**Published:** 2022-10-27

**Authors:** Md. Aktar-Uz-Zaman, Md. Ashraful Haque, Ashutosh Sarker, Md. Ashraful Alam, Md. Motiar Rohman, Md. Omar Ali, Mariam Abdulaziz Alkhateeb, Ahmed Gaber, Akbar Hossain

**Affiliations:** 1 Pulses Research Centre (PRC), Bangladesh Agricultural Research Institute (BARI), Ishurdi, Pabna 6620, Bangladesh; 2 Department of Genetics and Plant Breeding, Bangladesh Agricultural University (BAU), Mymensingh 2202, Bangladesh; 3 South Asia and China, International Centre for Agricultural Research in Dry Areas (ICARDA), New Delhi 110012, India; 4 Spices Research Center, Bangladesh Agricultural Research Institute Shibgonj, Bagura 5810, Bangladesh; 5 Plant Breeding Division, Bangladesh Agricultural Research Institute (BARI), Joydebpur, Gazipur 1701, Bangladesh; 6 Pulses Research Sub-Station, Bangladesh Agricultural Research Institute, Joydebpur, Gazipur 1701, Bangladesh; 7 Department of Biology, College of Science, Princess Nourah bint Abdlrahman University, P.O. Box 84428, Riyadh 11671, Saudi Arabia; 8 Department of Biology, College of Science, Taif University, P.O. Box 11099, Taif 21944, Saudi Arabia; 9 Division of Soil Science, Bangladesh Wheat and Maize Research Institute, Dinajpur 5200, Bangladesh

**Keywords:** heat-tolerant, heat-sensitive, lentil, screening, stress indices

## Abstract

Legumes, including lentil, are a valuable source of carbohydrates, fiber, protein and vitamins and minerals. Their nutritional characteristics have been associated with a reduction in the incidence of various cancers, HDL cholesterol, type 2 diabetes and heart disease. Among these quality parameters, lectins have been associated with reducing certain forms of cancer, activating innate defense mechanisms and managing obesity. Protease inhibitors such as trypsin and chymotrypsin inhibitors have been demonstrated to reduce the incidence of certain cancers and demonstrate potent anti-inflammatory properties. Angiotensin I-converting enzyme (ACE) inhibitor has been associated with a reduction in hypertension. Therefore, legumes, including lentils, should be part of our daily food intake. However, high temperatures at the terminal stage is a major abiotic constraint leading to a reduction in lentil yield and seed quality. Thus, the selection of heat-tolerant genotypes is essential to identifying the potential for high yields with stable performance. To select lentil genotypes, an experiment was conducted with 60 genotypes including local landraces, advanced breeding lines, commercial varieties and exotic germplasm under stress and non-stress conditions from 2019 to 2020. This study was followed by a subset study involving screening based on a few physicochemical parameters and reproductive traits along with field performances. Different tolerance indices (i.e., stress susceptible index (SSI), relative heat index (RHI), tolerance (TOL), mean productivity (MP), stress tolerance index (STI), geometric mean productivity (GMP), yield index (YI), yield stability index (YSI), heat-resistance index (HRI), modified stress-tolerance index (MSTI), abiotic tolerance index (ATI) and stress susceptibility percentage (SSPI)) were used for the selection of the genotypes along with field performance. Biplot analysis was further performed for choosing the most suitable indices. Based on principal components analysis, the GMP, MP, RRI, STI, YI, YSI, ATI and MSTI indices were identified as the most reliable stress indicators, and these indicators might be used for distinguishing heat-tolerant genotypes. Based on the stress indices, the genotypes BLX 05002-3, BLX 10002-20, LRIL-21-1-1-1-1, LRIL-21-1-1-1-1-6 and BLX 09015 were selected as the most stable and heat-tolerant genotypes. In contrast, the genotypes LG 198, Bagura Local, BLX 0200-08-4, RL-12-178, Maitree, 91517 and BLX 11014-8 were selected as the most heat sensitive. Data also exhibited an average yield reduction of 59% due to heat stress on the lentils. Moreover, eight heat-tolerant (HT) genotypes (BLX 09015, PRECOZ, LRL-21-112-1-1-1-1-6, BLX 05002-3, LR-9-25, BLX 05002-6, BARI Masur-8 and RL-12-181), and two heat-susceptible (HS) genotypes (BLX 12009-6, and LG 198) were selected from the screened genotypes and subjected to further analysis by growing them in the following year under similar conditions to investigate the mechanisms associated with heat tolerance. Comparative studies on reproductive function and physiochemical traits revealed significantly higher pollen viability, proline accumulation, relative water content, chlorophyll concentration and a lower membrane stability index in HT genotypes under heat stress. Therefore, these heat-tolerant genotypes could be used as the parents in the hybridization program for achieving heat-tolerant transgressive segregation.

## 1. Introduction

Lentil is a prehistorically domesticated crop and is one of the legumes consumed for food globally [1]. Worldwide, lentil production is 6.54 million tons with a cultivated area of 5 million hectares and an average yield of 1305 kg ha^−1^ [2]. The central origin of lentil is Central Asia, and a major share of production comes from Asia [2]. It is a major winter food legume in Bangladesh based on consumer preference, although grass pea ranks in the first position due to its area coverage and production [3]. Low temperatures are essential for lentil vegetative growth, but warm temperatures are required at the maturity stage. However, 18–30 °C has been reported as the optimum temperature for its ideal growth and crop production [4,5].

In Bangladesh, the lentil-sowing date is delayed due to late harvesting of the preceding crop, such as late sown Aman rice in the north-western part of Bangladesh, and early vegetable cultivation is increasing in the western part of Bangladesh. In most cultivated areas, the lentil reproductive stage, especially the grain-filling stage, suffers adversely from elevated temperatures [6]. Generally, crop-growth stages such as germination, vegetative growth, dry-matter partitioning, reproductive organ development, reproductive processes [7,8] and grain filling [9], along with grain quality [10,11], are interrupted by stresses. However, among the various developmental process of the plant, the reproductive and grain-filling stages are more vulnerable to temperature stress in legume crops [12,13,14,15]. For instance, the temperature rising for a substantial period decreased the grain weight linearly. A temperature of 15–25 °C is optimum at the reproductive stage for legume crops, especially lentils [13], peas [14], and chickpeas [15]. Pollen sterility was recorded at over 32 °C at the reproductive stage in chickpeas [16] and lentils [17]. The greater sensitivity of the reproductive stage to heat stress compared to the vegetative stage is mainly attributed to damage to male components, which are severely impacted as a result of the disruption of developmental as well as functional aspects, such as sucrose and starch accumulation in pollen grains [18]. However, a global 28% yield loss of lentils was recorded due to abiotic constraints where as 13% yield reduction has been recorded due to heat stress, cold and frost in South Asia, 8% in Sub-Saharan Africa and 13% in CWANA [19].

A combined package of genetic improvement and cultural practices can help minimize the detrimental effects of various abiotic stresses on agricultural productivity [18]. Genetic improvement deals with the development of cultivars that perform better under stressful environments (high temperature, drought and salinity) leading to better economic yields [20]. However, screening cultivars under field conditions against heat involves significant challenges owing to interactions with various environmental factors but multiple screenable traits are available for successful selection [21], although the selection process is more expensive [22]. The cultivars or varieties identified through selection as tolerant are more durable, and form an eco-friendly variety-improvement process for increasing the production of any crop against adverse environmental effects. This is why this screening program based on stress and non-stress environments was undertaken to uncover selection criteria for the identification of tolerant sources from our existing local and foreign cultivars of lentils. For instance, exploiting stresses using heat or drought tolerance indices has been suggested by many researchers for the identification of stress-tolerant genotypes, comparing yield performances in chickpeas in stress and non-stress environments [23,24]. Likewise, several quantitative drought-tolerance indices, such as the stress-tolerance index (STI), mean productivity (MP), geometric mean productivity (GMP), harmonic mean (HARM), and stress tolerance (TOL), have been used widely for the evaluation of genotypes with better stress tolerances, such as drought-stress tolerance, in many crops [13,25,26,27]. Siahsar et al. [28] reported that STI, GMP and HARM were the best indices for the selection of lentil lines under drought stress. In addition, the adaptation mechanism of a genotype to terminal drought and heat stress is a desirable strategy to minimize the economic impact of climate change on agriculture [29,30,31]. Therefore, these tolerance indices were used for selecting the superior genotypes among the studied genotypes of lentils under heat stress conditions for future lentil-breeding programs.

## 2. Materials and Methods

### 2.1. Materials of the Study

A total of 60 lentil genotypes consisting of commercial varieties, local landraces, advanced breeding materials and foreign germplasms were collected from the world’s largest collection center, the International Centre for Agricultural Research in Dry Areas (ICARDA), to be used in this screening program (Table S1). Following further investigation related to reproductive function and physicochemical traits associated with heat mechanisms, a subset of eight heat-tolerant genotypes (HT), and two heat-susceptible genotypes (HS) were evaluated in the following year under a similar environment and management practice (Table S2).

### 2.2. Location and Prevailing Weather Conditions

**Figure 1:**
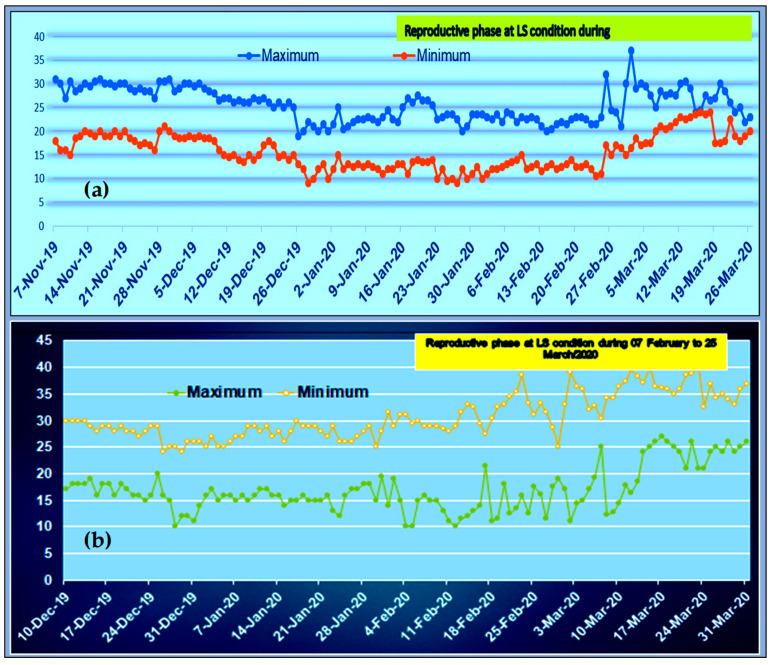
(**a**) The daily maximum and minimum temperature at the OS experimental field of lentils during growing seasons; (**b**): The daily maximum and minimum temperature at the LS experimental field (covered by polythene shed) of lentils during growing seasons.

This particular study was carried out at the research farm of PRC, BARI, Ishurdi, Pabna, Bangladesh. The daily maximum and minimum air temperature were recorded on the Zeal Maximum Minimum temperature scale under polythene shades and outside of the experiment to compare the raising temperature under the polythene shades and in control conditions during the crop season 2019–20 and 2020–21(Figure 1; Table S3). The rising temperature was recorded and the temperature under polythene shades was 3–4 °C higher compared to the non-stress condition during the whole crop cycle of the lentil plant (Figure 1).

### 2.3. Experimental Treatments and Design

In the 1st year, all collected genotypes were sown under two conditions: (1) one set was sown at the optimum sowing time on 7 November (OS) when day/night temperatures were within 32/8 °C (Figure 1) at their reproductive phase, and (2) another set was sown on 10 December (LS), (one month delay compared to the optimum sowing time) and the LS experimental plot was covered by polythene shades for raising the temperature for the whole life cycle of the crop. All treatments were arranged in an alpha-lattice design and repeated three times. The unit plot was 2.5 m long with two rows; the row-to-row distance was 30 cm with continuous seed sowing by hand. Seeds were placed at 3–5 cm depth and covered by surface soil. To ensure optimum germination, post-irrigation was applied just after sowing both sets of seeds, because the initial moisture of the soil was not sufficient. In the next season’s evaluation involving a reduced set of genotypes, the experiments (OS, LS) were laid out in a randomized complete block design in tree replication with 8 rows, 4 m long. Excluding the border rows, only the inner rows were used for physiochemical data collection, while only the middle 4 rows were used for collecting the yield and yield-contributing traits.

### 2.4. Observations Recorded

#### 2.4.1. First-Year Study

In the present study, consisting of sixty genotypes under OS and LS conditions, observations of several parameters were recorded. Days to flowering were counted from the sowing to the point at which 50% of flowers were visible in the plants on a plot basis. The canopy width of randomly selected plants was measured in cm in three positions of the planted rows. The height of five selected plants was measured in cm, representing the average height at the maturity stage. A hundred randomly selected seeds were weighed in grams on a plot basis. For the estimation of grain yield/plant, the whole plot yield divided by the total number of final plant stands was used, before storing the seeds at 8–10% moisture content. The straw yield was the average biomass yield of the five randomly selected plants after oven drying at 72 °C for three days.

The harvest index was counted using the following equation:

$$\mathrm{Harvest}\;\mathrm{Index}\;\left(\%\right)=\;\frac{Grain\;weight}{Biomass\;weight+Grain\;weigt}\times 100$$

#### 2.4.2. Estimation of Stress Tolerance indices

##### 2.4.2.1. Estimations of Heat-Tolerance Indices

Heat tolerance indices (stress susceptible index (SSI), relative heat index (RHI), tolerance (TOL), mean productivity (MP), stress tolerance index (STI), geometric mean productivity (GMP), yield index (YI), yield stability index (YSI), heat-resistance index (HRI), modified stress-tolerance index (MSTI), abiotic tolerance index (ATI) and stress susceptibility percentage (SSPI)) were estimated for the selection of the heat-tolerant genotypes and these heat-tolerant indices were calculated using the MS Excel program following these formulas:

1. Stress-susceptibility index $\left(\mathrm{SSI}\right)=\frac{1\;-\;\left(\frac{\mathrm{Ys}}{\mathrm{Yp}}\right)}{1\;-\;\left(\frac{\bar{Y}s}{\bar{Y}p}\right)}$ [32]
2. The relative heat index $\left(\mathrm{RHI}\right)=\frac{\left(\frac{\mathrm{Ys}}{\mathrm{Yp}}\right)}{\left(\frac{\bar{Y}s}{\bar{Y}p}\right)}$ [33]
3. Tolerance (TOL) = Yp − Ys [34]
4. Mean productivity $\left(\mathrm{MP}\right)=\frac{\mathrm{Ys}+\mathrm{Yp}}{2}$ [35]
5. Stress-tolerance index $\left(\mathrm{STI}\right)=\frac{\mathrm{Ys}\;+\;\mathrm{Yp}}{\left(\bar{Y}p\right)2}$ [36]
6. Geometric mean productivity $\left(\mathrm{GMP}\right)=\sqrt{\left(\mathrm{Yp}\right)\left(\mathrm{Ys}\right)}$ [37]
7. Yield index $\left(\mathrm{YI}\right)=\left(\frac{Ys}{\bar{Y}s}\right)$ [35]
8. Yield-stability index $\left(\mathrm{YSI}\right)=\left(\frac{Ys}{Yp}\right)$ [38]
9. Heat-resistance index $\left(\mathrm{HRI}\right)=\frac{\mathrm{Ys}\;\times \left(\frac{\mathrm{Ys}}{\mathrm{Yp}}\right)}{\bar{Y}s}$ [39]
10. Modified stress-tolerance index (MSTI) = K_1_STI, $k1=\frac{\left(Yp\right)2}{\left(\bar{Y}p\right)2}$ and $K2\mathrm{STI}=\frac{\left(Ys\right)2}{\left(\bar{Y}s\right)2}$ where ki is the correlation coefficient
11. Abiotic tolerance index $\left(\mathrm{ATI}\right)=\frac{\left(\mathrm{Yp}\;-\;\mathrm{Ys}\right)}{\left(\frac{\bar{Y}p}{\bar{Y}s}\right)}\times \sqrt{\left(\mathrm{Yp}\times \mathrm{Ys}\right)}$ [40]
12. Stress-susceptibility percentage (SSPI) = $\{\frac{\left(\mathrm{Yp}\;-\;\mathrm{Ys}\right)}{2\left(\bar{Y}p\right)}\}\times 100$ [40]

where Yp, Ys, p and s indicate the yield under normal sowing, yield under late sowing for each genotype, and mean yield in normal and late sowing conditions for all genotypes, respectively. For screening heat-tolerant genotypes, a rank-sum (RS) was calculated using the following relationship [41]:

Rank sum (RS) = Rank mean (R) + Standard deviation of rank (SDR)

##### 2.4.2.2. Estimations of Thermal Unit Indices

Different thermal unit indices, such as growing degree days (GDDs), helio-thermal units (HTU), pheno-thermal index (PTI) and heat-use efficiency (HUE), were calculated at the maturity stage according to Singh et al. [42]. GDDs were computed with 5 °C as the base temperature based on the daily mean temperature from the following equation:

$$\mathrm{GDD}\;\left(\mathrm{growing}\;\mathrm{degree}\;\mathrm{day}\right)=\frac{⅀\left(Tmax+Tmin\right)}{2}-\mathrm{Tb}$$

where Tmax = maximum temperature, Tmin = minimum max min temperature and Tb = base temperature of lentil.

HTUs (degree-days hours) of successive growth phases were calculated based on GDD and sunshine hours using the following formula:

HTU (degree-days hours) = [GDD] × Duration of sunshine hours

The duration of sunshine hours per day of successive growth phases was calculated by the following equation:

$$\mathrm{The}\;\mathrm{duration}\;\mathrm{of}\;\mathrm{sunshine}\;\mathrm{hours}\;=\frac{\mathrm{Total}\;\mathrm{bright}\;\mathrm{sunshine}\;\mathrm{hours}\;\mathrm{of}\;\mathrm{the}\;\mathrm{crop}\;\mathrm{stage}}{\mathrm{Duration}\;\mathrm{of}\;\mathrm{crop}\;\mathrm{stage}}$$

PTI (degree-days day^−1^) was calculated using the following equation:

$$\mathrm{PTI}\;\left(\mathrm{degree}-\mathrm{days}\;{\mathrm{day}}^{-1}\right)=\frac{\mathrm{GDD}}{\mathrm{Growth}\;\mathrm{day}}$$

Heat-use efficiency (HUE) (kg ha degrees-day) was calculated with the help of the following equation:

$$\mathrm{HUE}\;\left(\mathrm{kg}\;{\mathrm{ha}}^{-1}\;\mathrm{degrees}-\mathrm{day}\right)=\frac{\mathrm{Seed}\;\mathrm{yield}\;\left(\mathrm{kg}\;{\mathrm{ha}}^{-1}\right)}{\mathrm{GDD}}$$

#### 2.4.3. Physicochemical and Reproductive Trait Study of Screened Lentil Genotypes

Some physiological traits, such as proline content, chlorophyll content, relative water content (%), membrane thermal stability index, and one reproductive trait, pollen viability (%), were studied in the following year for further analysis to assess the tolerance mechanism with the association of rising temperature, and methodology of the assessment of these physiochemical traits is described in brief as follows:

##### 2.4.3.1. Chlorophyll Content (mg g^−1^ DW)

Before flowering, 0.1 g of fresh leaves from the control and stress treatment of each genotype were taken in an amber-colored bottle. Following this, 10 mL 80% acetone was mixed with the leaf sample and kept in a dark place for more than 24 h to dissolve the chlorophyll concentration following the procedure of Awasthi et al. [43]. After 24 h, the supernatant of leaves from the acetone solution was run in a spectrophotometer at 645 and 663 nm against 80% acetone as black. Total chlorophyll was then measured according to the following formula [44]:

$$\mathrm{mg}\;\mathrm{Chl}\;a/g\;\mathrm{tissue}\;=\left\{12.7\;\left(Abs663\right)-2.69\;\left(Abs645\right)\right\}\;\frac{V}{1000\times W}$$

$$\mathrm{mg}\;\mathrm{Chl}\;b/g\;\mathrm{tissue}\;=\left\{22.9\;\left(Abs645\right)-4.68\;\left(Abs663\right)\right\}\frac{V}{1000\times W}\;$$

mg Total chl/g tissue = Chl a + Chl b

where *A* = absorbance at a specific wavelength of the spectrophotometer; *V* = final volume of chlorophyll extract in 80% acetone; *W* = fresh weight of tissue extracted

##### 2.4.3.2. Proline Detection (mg g^−1^)

Proline content was detected following the ninhydrin method according to the procedure described by Bates et al. [45], which follows:

$$\mathrm{Proline}\;\left({\mathrm{mg}\;g}^{-1}\right)=\frac{Absorbance\;of\;sample\times K\;value\times dilution\;factor\;}{weight\;of\;sample\times 100}$$

##### 2.4.3.3. Relative Water Content (RLWC)

Relative leaf water content (RLWC) was determined following the methods of Barrs and Weatherley [46]. Before flowering, all fresh leaf samples from each control and stress genotype were collected and weighed (fresh wt). Leaves were then submerged in distilled water for 8 h in a Petri dish. After eight hours’ soaking, the water was removed from the Petri dish and the leaf samples were smoothed and surface dried with filter papers and weighed (turgid wt), then the leaf samples were oven-dried for up to 72 h at 70 °C and reweighed (dry wt). Finally, RLWC was calculated using the following equation:

$$\mathrm{RLWC}\;\left(\%\right)=\frac{Leaf\;Fresh\;wt-Leaf\;Dry\;wt\;}{Leaf\;Turgid\;wt\;–Leaf\;Dry\;wt}\times 100$$

##### 2.4.3.4. Membrane Thermostability Index (MSI)

The leaf membrane thermostability index (MSI) was measured following the protocol of Premachandra et al. [47] as modified by Sainnan [48]. For determination of leaf MSI, 2 mg fresh leaf of each genotype from control and stress and 20 mL double distilled water were taken in a Falcon tube. The Falcon tube was then placed in the water bath for boiling at 40 °C for 30 min. After that, the electrical conductivity (C_1_) of this boiling sample was measured by an electrical conductivity meter. Consequently, another sample of leaves of the same genotype was placed in a water bath for boiling at 100 °C for 10 min and electrical conductivity was recorded in the same way (C_2_). Finally, MSI was estimated with the following equation:

MSI = {1 − (C_1_/C_2_)}∗100

##### 2.4.3.5. Pollen Viability

During the days to flowering of each control and stress genotype, pollen grains were collected from opened flowers on the same days. The collected pollen grains were polled, and the section was prepared based on the size and shape of the pollen and stained with 0.5% acetocarmine/Alexander stain at 10 min [49]. The pollen grains were collected from flowers that opened on the same day. About 200 pollen grains were tested for pollen viability, and 5–10 microscopic field pictures were taken by stereo-microscope [50]. The collected pollen grains from the flowers were pooled and tested for their viability. To select viable pollen grains, the selection was made based on the size and shape (triangular or spherical) of the pollen, and the concentration of the stain taken up by the pollen [50].

### 2.5. Statistical Analysis

An analysis of variance of different yield and yield-contributing traits, principal component analysis (PCA), and a correlation study were analyzed by a statistical software program [51]. Additionally, a multicollinearity test was performed to discover the most suitable and powerful explanatory indices against the stress-tolerance genotypes using the same statistical software program [51].

## 3. Results

### 3.1. Screening Based on Field Performance

Sixty lentil genotypes were screened based on the field performance of different phenological and yield-contributing traits, such as days to flowering, plant height, canopy width, 100-seed weight, straw yield per plant, grain yield per plant and harvest index. Observations showed that in late-sown environments, all genotypes flowered earlier, ranging from 46 to 67, except genotype 7 (check variety BARI Masur-9) compared with optimum-sown conditions (range 36–73 days for flowering) (Table 1). Among all the genotypes, 55, 59, 29, 7, 8, 9, 53, 54 and 58 flowered early (45–52 days) under stress conditions, while genotypes 57, 47, 45, 46, 44, 40, 16, 48, 15, 23, and 41 flowered late (67–62 days). The average plant height of all genotypes was found to be shorter in optimum-sown (49.02 cm) compared to late-sown conditions (52.11 cm) except genotypes 8, 29, 32, 37, 41, 43, and 57. Canopy width was found to be narrow (28.18 cm) in late-sown compared to optimum-sown conditions (31.76 cm) in all genotypes except genotypes 10 and 11.

The hundred-seed weight (HSW) in OS plants ranged from 1.69–3.34 g, while LS plants ranged from 1.47–3.01 g (Table 2). In a stress environment, genotypes 16, 52, 59, 48, 58 and 41 produced the largest seeds (HSW 3.011–2.426 g) while genotypes 44, 11, 4, 1, 25 and 22 produced the lightest seeds (HSW 1.528–1.685 g) among all the genotypes. Grain weight per plant (GYP) in OS ranged from 0.68–3.57 g plant^−1^. In contrast, LS plants ranged from 0.22–1.37 g plant^−1^ (Table 2). In LS conditions, the genotypes 58, 54, 59, 29, 48, 9 and 8 produced the maximum GYP (1.369–0.940 g plant^−1^) while the genotypes 44, 57, 24, 47, 46 and 13 produced the minimum GYP (0.222–0.362 g plant^−1^) (Table 2). Straw yield per plant (SYP) in OS ranged from 1.54–4.99 g plant^−1^ while in LS plants this ranged from 0.83–3.14 g plant^−1^. In heat-stress conditions, genotypes 48, 57, 16, 22, 2 and 36 produced the maximum SYP (3.137–1.965 g plant^−1^) while genotypes 52, 8, 3, 43 and 7 produced the lowest SYP (0.828–1.031 g plant^−1^). The average harvest index (HI) ranged from 18.86–53.27% estimated in OS plants to 10.25–52.26% in LS plants. Among the genotypes 8 (52.26%), 54 (48.77%), 52 (48.28%), 29 (46.84%), 53 (45.48%) and 43 (41.92) gave the maximum HI, while the genotypes 57, 44, 46, 24, 25 and 40 showed the lowest, with 10.25%, 15%, 15.96%, 16.65%, 18.07% and 18.75% HI, respectively, in a heat-stress environment (Table 2).

### 3.2. Reduction in Grain Size and Grain Yield under Late-Sown Heat-Stress Conditions

**Figure 2:**
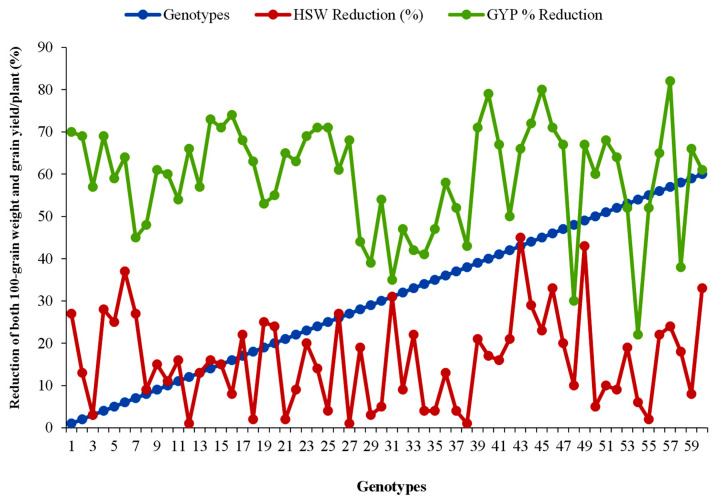
% Reduction in grain size and yield of 60 lentil genotypes in heat-stress conditions. HSW, 100-seed weight; GYP, grain yield/plant.

A wide range of variations in grain size and grain yield was revealed among the studied genotypes in both stress and non-stress environments. In LS plants, gain-size reductions of 1–45% were recorded. The maximum grain-size reduction of 45% was observed in genotype 43; the second-highest, 43%, in genotype 49; and the lowest reduction of 1% was observed in genotypes 12, 27 and 38 (Figure 2). Grain-yield reductions of 22–82% due to heat stress were recorded, and genotypes 57, 45, 40, 16, 14 and 44 showed maximum 82, 80, 79, 74, 73 and 72% grain-yield reductions compared to those under OS conditions. The minimum yield reduction of 22% was recorded from genotype 54 followed by genotypes 48, 31, 58 and 29 and the yield loss was estimated at 30, 35, 38 and 39%, respectively.

### 3.3. Ranking of Genotypes Based on Heat-Stress-Tolerance Indices

Significant yield variations were observed among all genotypes due to the terminal heat stress (Table 3 and Table 4).

The minimum yield reduction was recorded in genotypes 54, 59, 58, 9, 8, 16 and 29 under stress and non-stress environments compared to the other genotypes in the same condition, which means these genotypes were treated as terminal heat-stress tolerant. However, the maximum yield reduction was observed in genotypes 44, 57, 13, 24, 47 and 46. The calculated stress-tolerance attributes (Table 3) indicated that the selection of stress-tolerance genotypes based on a single selection criterion was incongruous. For instance, according to the TOL and SSI, genotypes 54 and 48 were the most desirable heat tolerance genotypes. According to RHI, genotypes 54, 48, 31, 58 and 29 and MP genotypes 59, 16, 58, 9 and 15 were the more relatively heat tolerance genotypes, respectively. According to STI and GMP, genotypes 59, 58, 16, 9, 29, 54, 53 and 15 were the most heat tolerant, whereas genotypes 44, 13, 24, 47, 57 and 12 were the least tolerant genotypes relatively. Following YI, genotypes 58, 54, 59 and 29 were the most—and 44, 57, 13 and 24 the least—tolerant genotypes relatively (Table 3 and Table 4). Based on the YSI and HRI, genotypes 54, 58, 48, 29 and 31 were the most yield-stable genotypes over the stress and non-stress environments. According to ATI and SSPI, genotypes 59, 16, 15 and 9 were the most tolerant genotypes relatively for stress environments with a low fluctuation of yield compared to the non-stress-environment yield performance. According to the K_1_STI, genotypes 59, 16, 58, 9 and 15—and for K_2_STI, genotypes 59, 58, 54, 29 and 15—were the most tolerant genotypes relative to the other studied genotypes.

The above-estimated indices of heat tolerance indicated that, based on a single index, the selection of heat-tolerant genotypes was more contradictory. Different indices indicated different genotypes as heat tolerant. For the determination of the most desirable heat-tolerant genotypes according to all studied indices’ mean rank (MR), the standard deviation of ranks (SDR) and rank-sum (RS) were estimated (Table 3 and Table 4). With consideration of all the indices, genotypes 54 (RS =13.55), 29 (RS = 17.55) 58 (RS = 19.73) and 8 (RS = 23.09) were selected as the most heat-tolerant genotypes. In contrast, genotypes 44 (RS = 69.00), 24 (RS = 65.51), 47 (RS = 64.29), 13 (RS = 64.19), 57 (RS = 62.75) and 45 (RS = 61.08) were identified the most sensitive to heat-stress environments among all the studied genotypes.

### 3.4. Multicollinearity Test

**Figure 3:**
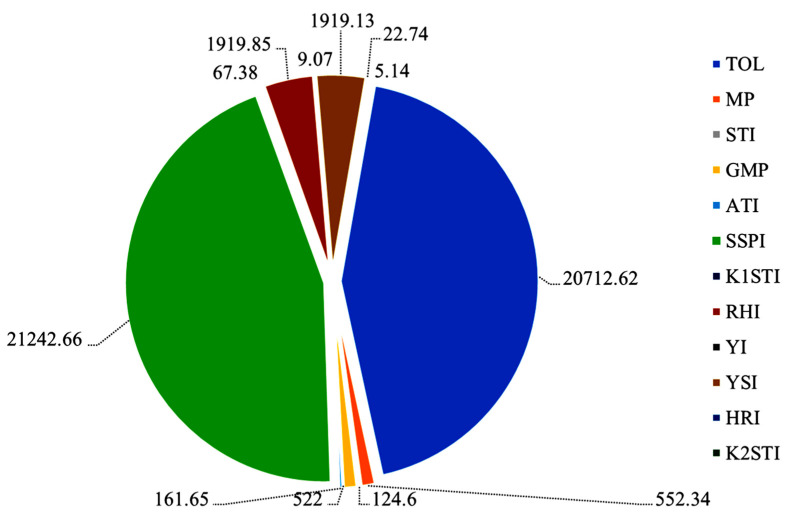
Multicollinearity diagnosis of Pearson product correlation matrix for 12 indices with their variance inflation factor (VIF). stress-susceptibility index (SSI), relative heat index (RHI), tolerance (TOL), mean productivity (MP), stress-tolerance index (STI), geometric mean productivity (GMP), yield index (YI), yield-stability index (YSI), heat-resistance index (HRI), modified stress-tolerance index (MSTI: K_1_STI, K_2_STI), abiotic tolerance index (ATI) and stress susceptibility percentage (SSPI).

A multicollinearity test was performed among the indices RHI, YI, YSI, HRI, K_2_STI, TOL, MP, STI, GMP, ATI, SSPI and K_1_STI (Figure 3). The results showed that the multicollinearity analysis varied from 67.37 to 21,242.66, and their variance inflation factor (VFI) values were >10. To find the more specific correlation among the indices, PCA was also performed.

### 3.5. PCA Component Analysis Method

**Figure 4:**
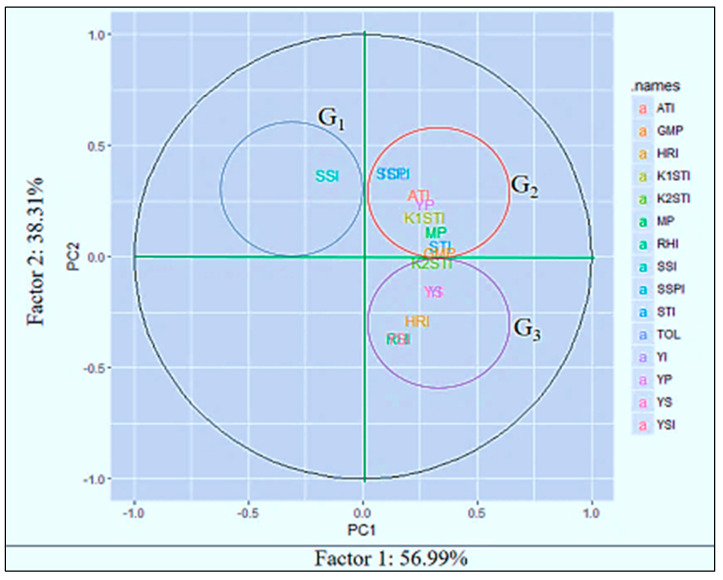
Biplot for lentil genotypes and stress indices using the first two principal components. Note: stress-susceptibility index (SSI), relative heat index (RHI), tolerance (TOL), mean productivity (MP), stress-tolerance index (STI), geometric mean productivity (GMP), yield index (YI), yield-stability index (YSI), heat-resistance index (HRI), modified stress-tolerance index (MSTI), abiotic tolerance index (ATI) and stress-susceptibility percentage (SSPI).

PCA is exploited as a “pattern-finding method” by plant breeders for completing cluster analyses [50]. The PCA method is more adventitious compared to cluster analysis and each statistic can be allocated to a single group only [51]. PCA1 and PCA2 are used for drawing a biplot that graphically represents the interrelationship among the different indices (Figure 4).

The first two PCAs explained more than 95% of the total variations. The PCA1 and PCA2 categorized the indices into different groups. From these studies, SSI was categorized as group 1 (G_1_). The PC axes separated Yp, TOL, SSPI, ATI, K_1_STI, MP and GMP in group 2 (G_2_), and Ys, YSI, RHI, HRI, YI and K_2_STI in group 3 (G_3_). “High heat tolerance” should be considered in terms of yield stability in a heat-stress environment. The genotypes that perform their yield with low fluctuations under different stress environments can be considered “highly heat tolerant”.

**Figure 5:**
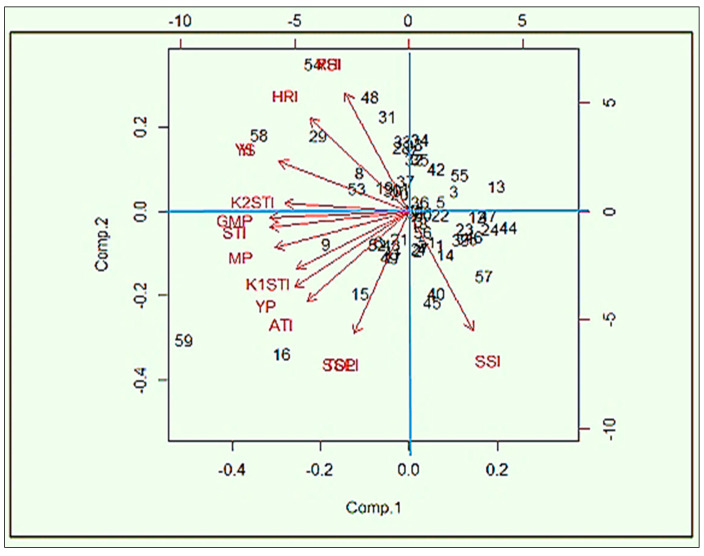
Biplot of lentil genotype and stress indices based on the first and second components of heat-tolerant indices. Note: stress-susceptibility index (SSI), relative heat index (RHI), tolerance (TOL), mean productivity (MP), stress-tolerance index (STI), geometric mean productivity (GMP), yield index (YI), yield-stability index (YSI), heat-resistance index (HRI), modified stress-tolerance index (MSTI), abiotic tolerance index (ATI) and stress-susceptibility percentage (SSPI).

From this study, HRI, K_2_STI, YI, and RHI might be used for screening “highly heat-tolerant” genotypes, as they have a strong association with the YSI (yield-stability index). In contrast, “heat-tolerance” should be considered based on acceptable yield performance under stress conditions and high-yield performance under normal environments and it not refer to the yield stability in both normal and stress environments. Thus, TOL, SSPI, ATI, K_1_STI, MP, STI and GMP might be considered tools for screening “heat-tolerant” genotypes as they have no relationship with YSI or their negative correlation. The path view of the biplot (Figure 5) represents a summary of the interrelationship among the heat indicators and also provides the importance of the indices, which is more or less emphasized for the identification of the individual index of the specific stress. Principal component analysis (PCA) showed that a significant positive correlation between grain yield in stress conditions with the TOL, SSPI, ATI, YP, K1STI, MP and GMP criteria; consequently, one can distinguish heat-tolerant genotypes with the same approach. Exploring the biplot diagram (Figure 4), genotypes 58, 59, 53, 54, 48, 31, 33, 28, 29, 19 and 8 were identified as tolerant and genotypes 9, 11, 14, 15, 16, 20, 22, 23, 24, 40, 44, 45, 47, 49, 56 and 57 were identified as sensitive to heat stress.

### 3.6. Correlation Study among the Stress Indices

A correlation among the study stress indices revealed that yield under a stress environment had a positive (r = 0.55) and significant (*p* ≤ 0.001) association with that of the normal environment (Table 5).

All stress indices had a positive and significant relationship with grain yield in the stress environment except SSI (r = −0.74, *p* ˂ 0.001), TOL (r = 0.03, *p* ˂ ns) and SSPI (r = 0.03, *p* ˂ ns) (Table 5). These results indicate GMP, MP, RHI, STI, YI, YSI, DI, ATI, K_1_STI and K_2_STI are the most significant and reliable selection indicators for the identification of stress-tolerant genotypes among the 60 genotypes of lentils studied.

### 3.7. Temperature Scenario and Thermal Unit Indices

The whole life cycle of a lentil is divided into different growth stages, such as seeding, vegetative, reproductive, and mature. The different thermal unit indices were measured at the maturity stage under optimum-sowing (OS), and late-sowing (LS) conditions (Table 6). The days to maturity of each genotype were greater in OS compared to LS condition. The days to maturity ranged from 93 to 119 days in OS, but in LS condition this ranged from 86–107 days. The yield performance of each genotype was also higher in the OS condition compared to the LS condition.

The GDD value is the maximum of each genotype in the LS condition compared to the OS genotypes, except the genotypes 2 and 14. Genotypes 15, 16, 18, 19, 37, 40, 45, 46, 51, 57 and 60 showed higher GDD in OS conditions. The received sunshine hours range was recorded at 6.21 to 6.48 h day^−1^ in OS, whereas in the LS condition this range was recorded from 5.90 to 6.15 h day^−1^. The helio-thermal units (HTU) of each genotype were recorded at a maximum in the LS condition compared to the OS except for some genotypes, and ranged was 480.43 to 660.51 in OS. These were recorded from 452.53 to 593.11 degree-day hours in LS condition. The pheno-thermal index (PTI) was recorded from 0.68 to 0.95 degree-day day^−1^ in OS and ranged from 0.82 to 0.97 degree-day day^−1^ in LS condition. The heat-use efficiency (HUE) of all genotypes under the OS condition was more or less similar (ranging from 41.42 to 42.82) but significant variance was recorded in LS condition and ranged from 5.72 to 30.26. The minimum HUE was recorded in the case of genotype 57, followed by genotypes 47, 46, 45 and 44. The maximum HUE (30.26) was recorded in genotype 33, followed by genotypes 29 (28.32), 59 (26.80), 58 (26.17), 9 (25.59) and 8 (25.44) with higher yield performance in OS and LS conditions.

### 3.8. Subset Study on HT and HS Genotypes

#### 3.8.1. Chlorophyll Concentration (mmol g^−1^ DW)

There was no significant variation among the studied genotypes in Chl a, Chl b and total Chl content. However, a reduction in chlorophyll was shown among all genotypes due to heat-stress conditions (Table 7). Numerically, the maximum total chlorophyll concentration (2.90 mmol g^−1^) was observed in the genotypes BARI Masur-8 followed by the genotypes LRL-21-112-1-1-1-1-6 and BLX 12009-6 and the minimum chlorophyll content 1.61 was observed in the genotype BLX 09015 under the control condition. In heat-stress conditions, the minimum total chlorophyll content of 0.94 mmol g^−1^ was observed in the genotype PRECOZ and the maximum total chlorophyll content of 1.56 was recorded in the genotype BARI Masur-8.

#### 3.8.2. Proline Concentration (mg g^−1^)

Among the different environmental stress conditions, solutes, such as proline, are normally accumulated in large quantities in higher plants for mitigating stress conditions, such as heat and drought. In this present study, proline content increased significantly under the late-sowing condition compared to the control condition due to the terminal heat-stress environment among all genotypes except the susceptible genotype LG 198. The maximum proline accumulation was found in the heat-tolerant genotype LR-9-25 followed by BLX-05002-6 and BLX-05002-3 and the minimum proline content was recorded in the susceptible genotype BLX 12009-6 (Table 8).

#### 3.8.3. Relative Water Content (%)

Under the control environment, RWC ranged from 76.67% to 83.67%, which was statistically non-significant, but in the stress condition, significant variations of RWC were recorded among all genotypes. The maximum RWC was recorded in the case of the tolerant genotype BLX 09015, with the minimum reduction percentage (7.17%), followed by the tolerant genotype BLX 05002-6 (7.50% reduction) and LR-9-25 (7.53% reduction) (Table 8). The highest reduction percentage (27.31%) of RWC compared to the respective control was recorded in the susceptible genotype LG-198 and followed by a 22.91% reduction in another susceptible genotype, BLX 12009-6.

#### 3.8.4. Membrane Thermostability Index (MSI)

The membrane thermostability index ranged from 12.00 to 15.53% among the genotypes in the OS condition, which increased from 41.89 to 95.70% due to heat stress in LS condition (Table 9). 

The maximum membrane thermostability index (27.73%) was recorded in the susceptible genotype LG-198 with the highest increasing rate (95.70%) followed by another susceptible genotype, BLX 12009-6 (25.67% MSI), with the second-highest increasing rate (84.28%) under the LS condition. The minimum MSI (17.77%) was recorded in the heat-tolerant genotype LRL-21-112-1-1-1-1-6 and followed by the tolerant genotypes BLX 05002-6 (18.10% MSI) and BLX 09015 (18.3%).

#### 3.8.5. Pollen Viability (%)

In the OS condition, the individual genotypes’ pollen viability ranged from 72.04 to 86.62%, which decreased by 3.34–21.60% in the terminal heat-stressed samples among all genotypes (Table 9). The maximum pollen viability reduction was recorded at 21.60% in the susceptible genotype LG-198 followed by 19.78% in another susceptive genotype, BLX 12009-6. The minimum pollen viability reduction of 3.34% was found in the heat-tolerant genotype BLX 09015 followed by the genotypes BLX 05002-6 (4.39%), LR-9-25 (4.71%), PRECOZ (7.45%) and BLX 05002-3 (7.50%).

## 4. Discussion

### 4.1. Screening Based on Stress Indices

In this present investigation, we imposed elevated temperatures on lentil genotypes by growing them one month later than the recommended date of sowing as well as covering late-sown experimental field with polythene shades, while a controlled plot was sown at the recommended sowing time for lentil crops. Creating high-temperature stress, delaying sowing, and covering the late-sown crops with polyethene shades is a worldwide recognized technique where the plants are grown in cooler weather to avoid high temperatures and drought stress during their reproductive development phase. However, twelve worldwide stress indices were used for the evaluation of these 60 lentil genotypes against high-temperature stress. Exploiting these indices indicated that the identification of heat-tolerant genotypes was more complex when based on a single index. Different indices indicated different genotypes as heat-tolerant; for instance, RHI and MP were found as suitable indices for identifying relatively more heat-tolerant genotypes, such as the genotypes 54, 48, 31, 58, and 29, and 59, 16, 9, and 15, respectively. However, the stress indices TOL and SSI were used for the selection of the most desirable heat-tolerant genotypes in this study, genotypes 54 and 48. On the other hand, the stress indices STI and GMP were used for screening the most heat tolerant and the least tolerant genotypes relatively, and selected the genotypes 59, 58, 16, 9, 29, 54, 53 and 15 as the most heat tolerant and 44, 13, 24, 47, 57 and 12 as the least relative tolerant genotypes. The index YI also indicated that the genotypes 58, 54, 59 and 29 were the most and 44, 57, 13 and 24 were the least tolerant genotypes relatively. On the other hand, TOl, ATI, SSPI and YI indices can be used for the selection of genotypes based on yield stability in the target environment [38]. For instance, Gavuzzi et al. [37] proposed YI as a significant positive index for discriminating genotypes by yield under stress conditions. Similar research findings were reported by Rosielle and Ramblin [35] and Fernanez [36]. They demonstrated that GPM is a powerful tool for separating genotypes by comparing with stress and non-stress environments. STI was used as a moderately heritable index for the selection of high-yielding genotypes in stress and non-stress conditions. Talebi et al. [52] reported that STI, GMP and MP indices could be capable of identifying high-yielding cultivars in both stress and non-stress environment. Puri et al. [53] reported that HTI, GMP and MP could be used as reliable selection criteria for terminal heat-tolerance spring-wheat genotypes in Nepal, whereas utilization of these stress indices for the selection of heat-tolerant genotypes in lentil is rare to date worldwide. From this study, we identified genotypes 44, 57, 13, 24, 47 and 46 as the maximum yield-reduction genotypes. However, genotypes 54, 59, 58, 9, 8, 16 and 29 were selected as yield-stable genotypes with a low fluctuation of yield loss under stress and non-stress environments compared to the other genotypes in the same condition. Considering the mean rank (MR), the standard deviation of ranks (SDR) and rank-sum (RS) of all studied indices to determine the most desirable heat-tolerant genotypes, the genotypes 54, 58 and 8 were selected as the most heat-tolerant genotypes. In contrast, genotypes 44, 24, 47, 13, 57 and 45 were selected as the most sensitive to heat stress environments among all the studied genotypes.

### 4.2. Screening Based On-Field Performance

Under field conditions, 60 lentil genotypes were sown one month later than the recommended sowing date and this late-sown experimental plot was covered by polythene shades to artificially create a high temperature. Another control experimental plot was sown at the recommended sowing date with the same genotypes, experimental design and management practices without polyethene shades. The temperature was always 3–4^0^ C higher in LS experimental plots than in OS experimental plots. A very limited set of experiments using the late sowing of lentils covered by polyethene shades has been conducted under field conditions globally. To date, one experiment has been performed by Delahunty et al. [54] in Australia, using delayed sowing with polyethene shades, but sowing crops late than the recommended time has been used globally for screening high-temperature-tolerant genotypes of lentil [55] and chickpea [23,56,57] by Kumar et al. [57] and of mungbean by Sharma et al. [58]. 

In this present study, a significant detrimental effect of high temperature was revealed in the case of yield and all yield-contributing traits except on branches per plant in the LS environment. High temperature has a detrimental effect on growth and reproductive physiology due to lentil’s narrow gene pool [59]. Days to flowering and days to maturity in the LS environment were significantly reduced in all genotypes of lentil except genotype 7 (BARI Masur-9), which flowered late under LS conditions. Genotype 7 (BARI Masur-9) is a short-duration variety that has the capability to escape terminal heat stress. Krishnamurthy et al. [23] reported that high temperature stimulates the flowering and reduces the days to maturity in chickpea, which partially supports the present study in lentil. High temperatures increased the plant height in all genotypes of lentil in LS conditions compared with OS condition, except in genotypes 1, 8, 28, 29, 30, 32, 37, 43, 46 and 57, but reduced canopy width was recorded in the case of all genotypes in the LS environment except genotypes 10 and 11, which indicates that high temperatures enhance the longitudinal expansion of plant cells in lentil. Winter-loving legume pulses are especially sensitive to an elevated temperature at their flowering and pod-development stages. At these stages, if >30 °C temperature was continued for a few days serious yield loss was reported, owing to flower drop and pod abortion [55,60,61,62]. Despite this, this sensitivity to high temperature varies from genotype to genotype. In this investigation, the temperature during the reproduction stage of lentils in the LS environment was above the threshold level (average maximum temperature was >33 °C, Appendix III), which indicated that a suitable environment was created in the LS environment for screening high-temperature-tolerant lentil genotypes. A similar environmental condition was also created by Kumar et al. [57] in lentils and by Krishnamurthy et al. [23] in chickpeas for the field screening of high-temperature-tolerant genotypes. Filled pods per plant were significantly decreased in the LS condition compared with the OS condition in the case of all genotypes in this present study but the decreasing rate of filled pods plant^−1^ was comparative low in the genotypes 58, 10, 35, 2, 4 and 18. Unfilled pods per plant were significantly higher in the OS condition compared to the LS condition in all studied genotypes; this occurred due to maximum pod abortion in the LS environmental condition as a result of the detrimental effects of high temperature in the lentil’s reproductive stage. Early studies showed that reproductive growth is more penetrating and causes various effects, such as the depletion of buds, flowers, fruits, pods and seeds, resulting in marked reductions in yield potential [63,64]. A significant reduction in grain size and grain yield was observed among the studied genotypes in LS compared with OS environment. On average, 16% gain-size deduction and 59% grain-yield reduction were found among the genotypes in the present investigation. Genotypes 57, 45, 40, 16, 14 and 44 were identified as the most heat-susceptible genotypes and the grain-yield reduction was recorded to be 82, 80, 79, 74, 73 and 72%, respectively, compared to the OS condition. A similar study was performed by Noureddine et al. [65] in lentil and 69.65% yield reduction was observed for heat stress only, and an 83% grain yield loss for a combined heat- and drought-stress environment. Kumar et al. [57] also recorded an average 2.4% to 67.2% grain-size reduction in late sowing conditions over the normal sown conditions in lentils. In our present investigation, grain-size reduction was associated with a reduction in the seed-filling rate and duration. Earlier reports indicated that a decrease in grain weight in response to heat or drought stress during early grain filling may be assigned primarily to a decline in the number of endosperm cells [66]; whereas during later grain filling, to a disturbance of starch synthesis due to the decreased availability of assimilates for developing seeds [67] or direct inhibitory effects of these stresses on the synthesis of storage substances [68]. The seed development stage of all grain crops is a critical growth stage under heat or drought stress and heat stress affects the seed filling adversely by suppressing the transfer of essential assimilates, exacerbating low grain yields and poor grain quality [9]. Due to elevated heat stress, yield reduction was documented in many cultivated crops, including cereals (rice and wheat), pulses (e.g., chickpea, cowpea), and oil crops (mustard, canola) [69,70,71,72]. For every 1 °C increase in seasonal temperature, grain yield in rice was decreased by 4.1 to 10.0% [73]. Late sowing and temperatures over 28–30 °C caused 53–73% yield loss in wheat [74]. In sorghum, a 53% reduction of filled seed weight and a 51% reduction in seed size was recorded due to heat stress, which exacerbated yield loss [75]; in mustard 52% yield reduction was recorded at a high temperature of over 30 °C [76]. In common beans, 26–37% yield reduction was recorded compared to non-stress conditions [77].

### 4.3. PCA Component Analysis Method

PCA is exploited by plant breeders as a “pattern finding method” for completing cluster analysis [50]. The PCA method is more adventitious compared to cluster analysis and each statistic can be allocated to a single group only [51]. A biplot was drawn based on the PCA1 and PCA2 components, which graphically represented the interrelationship among the different indices. More than 95% of the total variations could be explained based on the first two PCAs. The PCA1 and PCA2 also categorized the indices into different groups. Among the twelve indices, the SSI is categorized as group 1(G_1_), Yp, TOL, SSPI, ATI, K_1_STI, MP and GMP in group 2 (G_2_), and Ys, YSI, RHI, HRI, YI and K_2_STI in group 3 (G_3_). “High heat-tolerance” should be considered in yield stability with low fluctuations under different stress environments. The indices HRI, K_2_STI, YI and RHI could be used for screening “highly heat-tolerant” genotypes as they have a strong association with YSI (yield-stability index). In contrast, “heat-tolerance” should be considered based on acceptable yield performance under stress conditions and high yield performance under normal environments and it should not refer to the yield stability in both normal and stress environments. Thus, TOL, SSPI, ATI, K_1_STI, MP, STI and GMP could be considered tools for screening “heat-tolerant” genotypes as they have no relationship with YSI or their negative correlation. The path view of the biplot represents a summary of the interrelationships among the heat indicators and also provides the importance of the indices, which is more or less emphasized for the identification of the individual index of the specific stress. Principal component analysis (PCA) showed that a significant positive correlation was observed between grain yield in stress conditions with the criteria TOL, SSPI, ATI, YP, K1STI, MP and GMP; consequently, they can distinguish heat-tolerant genotypes with the same approach. Exploring these indices for the selection of heat-tolerant genotypes in lentils is very rare globally but very common in durum and bread wheat [39,46] for the screening of drought-tolerant genotypes under water-stress conditions. Similarly, HTI, GMP and MP indices were identified as trustworthy selection indicators for terminal heat-tolerance spring-wheat genotypes in Nepal [78]. Mohammadi et al. [78] and Sareen et al. [79] also reported similar findings in other environments for screening selection criteria of terminal heat-tolerant indices. The biplot diagram was used for the screening of tolerant and sensitive genotypes from this study. The genotypes 58, 59, 53, 54, 48, 29, 31 and 19 were identified as tolerant, and in contrast the genotypes 9, 11, 14, 15, 16, 45, 40, 57, 56 and 49 were perceived as sensitive to heat stress. A similar study was performed by Assefa et al. [80] for the identification of drought-tolerant bread-wheat genotypes.

### 4.4. Multicollinearity Test

Multicollinearity tests are used as a powerful tool by plant breeders to find whether multicollinearity exists between measured traits or indices [81,82]. The results from the present investigation showed that multicollinearity was observed among the indices GMP, YSI, K_1_STI and K_2_STI. Thus, these indices could be used for the identification of high-heat-tolerance genotypes based on the stress-tolerance yield performance of the 60 studied lentil genotypes. Many researchers have used multicollinearity better ascertain relationships between interpretive traits and yield performance [83,84].

### 4.5. Correlation Study among the Indices

A correlation study among the studied indices revealed that GMP, MP, RDI, STI, YI, YSI, DI, ATI, K_1_STI and K_2_STI were the most significant and reliable selection indicators for the screening of stress-tolerant genotypes among the 60 studied genotypes of lentil. A similar finding was recorded by Puri et al. [52] in spring wheat in Nepal.

### 4.6. Temperature Scenario and Thermal Unit Indices

Temperature, as well as sunshine hours, are significant and basic weather elements for any crop’s growth and development. For development from one growth stage to another, the crop needs a specific time and amount of heat (measured in terms of growing degree days, GDD). In the present study, the 60 lentil genotypes were evaluated based on the different thermal unit indices with their days to maturity and yield performance in OS and LS conditions. In the case of yield performance, each genotype produced a higher yield in the OS condition compared to the LS condition. All studied genotypes showed a higher GDD in LS condition except genotypes 2, 14, 15, 16, 18, 19, 37, 40, 45, 46, 51, 57 and 60. The received sunshine duration of each genotype in hours was maximal in OS compared to LS conditions. The helio-thermal units (HTU), and the pheno-thermal index (PTI) were recorded at their maximum in OS compared to LS conditions for each genotype. The heat-use efficiency (HUE) was more or less stable in each genotype under the OS condition but in LS conditions the HUE declined in the case of all genotypes except genotypes 29, 33, 58, 59, 9 and 8 due to their higher yield performance. Genotypes 29, 33, 58, 59, 9 and 8 were identified as heat-tolerant genotypes based on the thermal unit indices, whereas genotypes 57, 47, 46, 45 and 44 were selected as heat-sensitive genotypes due to their lower yield performance. Similar research was conducted by Islam et al. [85] in the case of mustard varieties. Several previous studies were conducted by Srivastava and Balkrishna [86], Khushu et al. [87] and Singh et al. [42], who indicated that thermal indices influenced crop phenological phases.

### 4.7. Subset Study on HT and HS Genotypes

#### 4.7.1. Chlorophyll Concentration

The stability of the chlorophyll concentration in plant cells during stress conditions is reported as a potential tool for selection against stressful environments in peanuts [88] and pigeon peas [89]. Chlorophyll concentration was decreased among all tolerant and susceptible genotypes but the concentration reduction was lower in tolerant genotypes compared to the susceptible genotypes. Reduction in chlorophyll concentration occurred in the stress environment, due to the disturbance of chloroplast membranes by direct or indirect effects such as photo-oxidation, which denatures the chlorophyll molecules [90,91,92]. Similar findings have been reported by Dash et al. [93] and Sehgal et al. [8] in lentils. The reduction rate of chlorophyll concentration in the leaves has been also reported more due to drought stress compared to heat stress due to the impact on chlorophyll fluorescence [8].

#### 4.7.2. Proline Content

The genotype LR-9-25 accumulated a higher proline content followed by the genotypes BLX-05002-6 and BLX-05002-3, and the minimum concentration was recorded in the case of the susceptible genotype BLX-12009-6. However, proline accumulations of the studied genotypes were found to be at their maximum in the stress environment compared to those sown at the optimum time. These results clearly indicate that the genotypes LR-9-25, BLX 05002-6 and BLX 05002-3 are more tolerant than the genotypes BLX 12009-6 and LG-198 owing the higher proline concentration under stress conditions. A similar finding was observed by Stoyanov [94] in beans, where susceptible cultivars accumulated a higher content of proline, and the proline content also acted on the reduction in RWC in the plant under water-stress conditions. Increasing proline content in leaves with decreasing water supply in higher plants indicates an efficient mechanism for osmotic regulation. The stabilization of sub-cellular structures and cellular adaptation to water stress occurred in maize [95] and chickpea [96]. In addition, proline acts as an osmolyte for osmotic adjustment, helping to stabilize sub-cellular structures (e.g., membranes and proteins), scavenging free radicals, buffering cellular redox potential, and maintaining the structure of the enzyme and removal of reactive oxygen species under stressed conditions [97].

#### 4.7.3. Relative Water Content (RWC)

The relative water content (RWC) is the percentage of water held by plant tissues. Generally, RWC is at a maximum in plant tissue in control environments compared to stress conditions. From this study, no significant variations were observed in any lentil genotypes during the control condition, but all genotypes exhibited a significant reduction in relative water content during the terminal heat-stress condition, and this reduction was significantly greater in susceptible genotypes than in the tolerant ones. A similar result was observed by Dash et al. [93]. The reduced RWC was also observed by Islam et al. [98], Nazran et al. [99] in mungbean, and Pospíšilová et al. [100] in French beans due to a water-stress environment.

#### 4.7.4. Membrane Thermostability Index (MSI)

Elevated temperature stress affects a plant’s normal physiological parameters adversely, such as membrane stability, photosynthesis, respiration and protein metabolism, which exacerbates low productivity in growth and yield [101]. Several research findings from previous scientists have also confirmed that the membrane thermostability of plant cells is an important factor for screening heat-tolerant genotypes [102,103]. Hence, the MSI has been increased in heat-stress conditions compared to tolerant genotypes, indicating significant membrane damage occurred in the susceptible lentil genotypes in the present investigation. A similar research finding was reported by Almeselmani et al. [104] in wheat genotypes, where a significantly increased membrane-injury index was observed in all genotypes under high temperatures and LS environments.

#### 4.7.5. Pollen Viability

The reproductive stages, especially the flowering and grain-filling stages, are more susceptible to elevated temperature stress; a few degrees of rise in temperature may lead to complete crop loss during the pollen-development stage [105,106,107]. The research findings of Kaushal et al. [18], Jiang et al. [108] and Sage et al. [109] indicate that the pollen viability of legume crops is more susceptible to high-temperature stress interrupting the reproductive function by altering the concentrations of phytohormones such as abscisic acid [110] and auxins [111]. However, in this present investigation, all lentil genotypes received more than 32 °C temperature during their reproductive stages, such as the pollen-development stage, due to the high temperatures imposed by the polythene shades and LS conditions (Table S3). In this study, the reduction in pollen viability was higher in susceptible genotypes compared to the tolerant genotypes, which may have reduced fertilization leading to flower abortion [16,112]. A similar finding was reported by Sita et al. [20].

## 5. Conclusions

Stress indices are successfully used for the identification of superior genotypes with tolerant genetic resources and better agronomical performance in a stressful environment. However, twelve stress indices were explored in this present investigation and among these indices, GMP, MP, RDI, STI, YI, YSI, DI, ATI, K_1_STI and K_2_STI have been identified as the most significant and reliable selection indicators for the evaluation of heat-tolerant genotypes based on comparing the performance of the 60 lentil genotypes under stress and non-stress environments. With consideration of all the indices, genotypes 54(BLX 10002-20), 29(BLX 05002-6), 58(BLX 05002-3) and 8(LRIL-21-67-1-1-1-1) were selected as the most heat-tolerant genotypes. In contrast, genotypes 44 (Bagura local), 24 (BLX 0200-8-4), 47 (Maitree), 13 (91517), 57 (LG 198) and 45 (RL-12-178) were identified as the most sensitive to heat-stress environments among all the studied genotypes. Simultaneously, considering the field performance under stress and non-stress environments indicated that the genotypes 54 (BLX 10002-20), 58 (BLX 05002-3) and 29 (BLX 05002-6) showed the maximum harvest index, with a minimum yield reduction of 22, 38 and 39%, respectively, in the heat-stress environment. In contrast, genotypes 57(LG 198), 40 (BLX 12004-5) and 44 (Bagura local) showed the minimum harvest index with the maximum yield reductions of 82, 79, and 72%, respectively. The average yield loss due to heat stress in lentils was recorded at 59% in this present investigation.

## Figures and Tables

**Table 1:** Phenology of 60 lentil genotypes under optimum- and late-sown conditions. The mean ± SE for each genotype in both conditions was calculated from three replications.

Genotypes	Days to Flowering		Plant Height		Canopy Width	
	OS	LS	OS	LS	OS	LS
1	66 ± 0.50	56 ± 0.50	54.03 ± 0.60	47.50 ± 1.90	30.54 ± 0.34	28.85 ± 0.67
2	65 ± 1.00	56 ± 0.50	43.88 ± 1.00	49.27 ± 1.10	34.68 ± 0.50	30.23 ± 2.00
3	65 ± 1.00	58 ± 0.00	45.62 ± 1.00	47.41 ± 3.20	30.82 ± 2.67	26.80 ± 1.17
4	66 ± 1.50	57 ± 1.50	49.10 ± 0.70	57.68 ± 1.40	32.82 ± 3.00	28.85 ± 0.67
5	65 ± 0.50	58 ± 1.50	45.71 ± 1.00	51.57 ± 0.30	32.25 ± 1.67	27.21 ± 1.00
6	67 ± 1.71	55 ± 1.00	49.58 ± 1.37	51.92 ± 0.70	31.68 ± 1.14	27.90 ± 1.17
7	36 ± 1.00	49 ± 1.50	41.85 ± 1.20	45.99 ± 0.80	25.53 ± 0.83	21.18 ± 0.33
8	52 ± 1.00	51 ± 1.50	53.64 ± 0.40	52.37 ± 0.40	31.11 ± 0.33	26.94 ± 2.33
9	61 ± 0.50	51 ± 1.00	46.39 ± 0.30	52.63 ± 0.50	30.54 ± 0.33	28.03 ± 1.67
10	66 ± 0.50	55 ± 1.50	50.26 ± 0.50	54.49 ± 1.20	29.97 ± 1.00	31.45 ± 2.17
11	68 ± 0.50	57 ± 0.50	51.42 ± 0.30	53.78 ± 0.20	28.39 ± 0.50	29.40 ± 1.00
12	66 ± 1.00	61 ± 1.50	48.03 ± 0.80	52.01 ± 1.40	30.68 ± 1.67	30.64 ± 0.50
13	67 ± 2.00	61 ± 1.50	49.97 ± 0.60	51.04 ± 1.00	33.68 ± 1.33	31.18 ± 2.00
14	67 ± 0.50	55 ± 0.00	45.91 ± 0.60	55.46 ± 0.30	35.11 ± 1.00	31.04 ± 1.67
15	69 ± 1.00	62 ± 1.50	51.71 ± 2.60	52.81 ± 0.10	34.83 ± 1.33	30.09 ± 0.17
16	68 ± 0.50	64 ± 1.00	53.45 ± 1.00	55.64 ± 0.50	32.97 ± 0.50	28.85 ± 1.00
17	69 ± 1.00	57 ± 1.00	47.07 ± 1.20	47.06 ± 0.60	31.11 ± 0.33	27.76 ± 1.00
18	67 ± 0.50	59 ± 0.50	46.49 ± 1.40	52.63 ± 1.10	32.25 ± 0.33	29.52 ± 0.49
19	69 ± 1.00	58 ± 1.00	45.91 ± 0.20	48.74 ± 1.50	32.39 ± 0.50	27.90 ± 0.17
20	66 ± 0.00	54 ± 2.00	46.68 ± 0.20	56.88 ± 2.70	34.97 ± 0.83	26.80 ± 0.17
21	67 ± 0.00	57 ± 1.50	44.84 ± 1.10	51.22 ± 0.70	33.40 ± 2.00	27.76 ± 0.34
22	67 ± 2.50	64 ± 0.00	51.13 ± 0.20	52.37 ± 2.40	31.82 ± 1.83	29.27 ± 1.17
23	68 ± 1.50	62 ± 2.00	51.32 ± 0.80	53.07 ± 0.80	33.68 ± 2.67	28.31 ± 0.67
24	69 ± 1.00	56 ± 1.00	51.32 ± 0.60	54.05 ± 0.70	31.11 ± 0.67	29.68 ± 1.00
25	71 ± 0.00	62 ± 0.50	49.77 ± 1.00	56.97 ± 1.80	34.68 ± 0.17	28.44 ± 0.17
26	65 ± 0.00	59 ± 1.00	46.97 ± 0.50	55.91 ± 2.20	28.82 ± 2.00	27.21 ± 1.00
27	64 ± 0.50	56 ± 1.00	42.33 ± 1.50	48.38 ± 0.10	32.68 ± 0.83	27.35 ± 1.67
28	65 ± 0.50	56 ± 0.50	51.03 ± 0.30	50.86 ± 0.10	32.96 ± 1.67	30.50 ± 0.67
29	64 ± 0.50	50 ± 1.00	59.15 ± 1.30	50.33 ± 0.30	34.11 ± 0.50	29.95 ± 0.33
30	66 ± 0.50	52 ± 1.00	48.71 ± 0.10	53.34 ± 1.30	32.11 ± 0.17	30.77 ± 1.00
31	65 ± 0.50	55 ± 0.00	44.36 ± 1.60	54.14 ± 0.80	32.96 ± 0.83	30.77 ± 0.67
32	66 ± 2.00	55 ± 0.00	49.39 ± 0.80	48.38 ± 0.50	33.39 ± 0.99	30.50 ± 0.67
33	66 ± 0.50	54 ± 1.00	45.13 ± 0.40	52.99 ± 0.90	34.97 ± 0.17	32.00 ± 0.50
34	66 ± 2.50	57 ± 1.50	48.13 ± 1.90	53.69 ± 1.10	33.40 ± 2.00	30.36 ± 1.83
35	64 ± 0.50	56 ± 1.00	51.90 ± 1.40	51.04 ± 0.70	33.11 ± 0.33	29.68 ± 0.67
36	66 ± 0.50	59 ± 1.00	44.36 ± 0.60	50.15 ± 0.50	29.82 ± 0.50	26.39 ± 1.00
37	66 ± 0.50	54 ± 1.00	44.17 ± 1.00	43.87 ± 2.00	32.11 ± 0.50	25.29 ± 0.34
38	66 ± 1.00	55 ± 1.00	44.94 ± 1.00	47.23 ± 0.20	33.25 ± 0.17	29.13 ± 0.67
39	65 ± 0.50	54 ± 1.00	50.26 ± 0.50	57.15 ± 0.20	33.39 ± 1.34	27.07 ± 0.50
40	70 ± 1.00	65 ± 0.50	46.29 ± 0.40	46.79 ± 0.70	30.39 ± 0.84	28.85 ± 0.33
41	65 ± 1.00	62 ± 2.00	63.11 ± 1.60	49.45 ± 0.50	34.83 ± 1.34	26.94 ± 0.33
42	66 ± 0.50	59 ± 1.00	51.80 ± 1.30	53.07 ± 1.20	33.53 ± 1.50	27.08 ± 1.84
43	65 ± 0.50	55 ± 1.00	48.32 ± 1.50	47.72 ± 0.75	30.53 ± 1.00	26.66 ± 0.33
44	69 ± 1.00	65 ± 0.50	46.87 ± 0.80	46.26 ± 2.90	32.54 ± 1.00	26.80 ± 0.50
45	66 ± 0.50	66 ± 1.50	53.64 ± 0.20	59.36 ± 6.50	31.97 ± 1.33	26.39 ± 0.67
46	73 ± 1.00	66 ± 1.00	64.76 ± 0.90	56.00 ± 0.30	33.39 ± 0.34	26.52 ± 1.67
47	70 ± 0.50	67 ± 1.50	55.28 ± 1.30	60.07 ± 0.90	29.96 ± 0.67	26.11 ± 0.67
48	70 ± 0.00	63 ± 1.00	53.35 ± 1.50	59.36 ± 1.30	30.54 ± 0.67	28.17 ± 0.83
49	66 ± 0.50	56 ± 0.00	49.87 ± 0.70	52.37 ± 2.20	31.68 ± 0.67	29.81 ± 1.17
50	66 ± 1.50	57 ± 2.00	46.58 ± 0.50	54.67 ± 0.40	28.82 ± 1.00	28.03 ± 0.33
51	67 ± 0.00	57 ± 0.50	45.62 ± 0.50	54.93 ± 1.70	28.82 ± 1.34	26.25 ± 0.83
52	72 ± 0.50	53 ± 4.50	39.62 ± 1.50	44.67 ± 2.90	29.11 ± 0.33	25.70 ± 2.83
53	69 ± 0.50	51 ± 0.50	40.11 ± 1.60	46.61 ± 0.30	27.82 ± 0.50	26.39 ± 1.00
54	70 ± 1.00	51 ± 0.50	38.66 ± 0.50	49.27 ± 0.70	26.82 ± 1.00	24.47 ± 1.00
55	67 ± 1.00	45 ± 0.50	46.10 ± 1.00	47.41 ± 1.40	31.97 ± 1.00	28.03 ± 1.00
56	66 ± 1.00	57 ± 0.50	56.64 ± 0.50	60.24 ± 0.90	32.97 ± 0.50	27.48 ± 1.00
57	71 ± 1.50	67 ± 1.00	64.47 ± 0.80	59.01 ± 1.90	32.25 ± 0.67	26.80 ± 2.17
58	60 ± 1.00	52 ± 2.50	50.84 ± 1.70	50.24 ± 1.60	31.82 ± 1.83	28.03 ± 0.00
59	53 ± 1.00	46 ± 0.00	48.90 ± 0.50	57.32 ± 1.80	24.82 ± 1.00	26.80 ± 0.17
60	64 ± 1.50	56 ± 1.00	44.46 ± 1.50	49.76 ± 0.95	32.68 ± 1.70	28.58 ± 0.00
Heritability	0.97	0.95	0.97	0.89	0.86	0.82
Mean	65.71	57.04	49.02	52.11	31.76	28.18
Range (mean)	36.00–73.00	45.00–67.00	41.85–64.76	43.87–60.24	24.82–35.11	21.18–32.00
SE±	0.828	1.025	0.876	1.167	0.936	0.880
Range (S.E)	0.00–2.50	0.00–4.50	0.20–2.60	0.20–3.20	0.17–3.00	0.17–2.83
LSD (0.05)	1.91 **	2.37 **	1.99 **	2.93 **	1.85 **	1.81 **
CV (%)	2.09	3.02	2.92	4.22	4.45	5.00

** *p* ≤ 0.001; ns., not significant; OS, optimum sowing and LS, late sowing.

**Table 2:** Yield and yield-contributing traits of 60 lentil genotypes under optimum and late sown. The mean ± SE for each genotype in both conditions was calculated from three replications.

Genotypes	HSW		GYP		SYP		HI	
	OS	LS	OS	LS	OS	LS	OS	LS
1	2.303 ± 0.07	1.685 ± 0.09	1.621 ± 0.06	0.494 ± 0.04	4.979 ± 0.20	1.440 ± 0.00	25.48 ± 0.85	25.29 ± 1.43
2	2.174 ± 0.19	1.900 ± 0.20	1.846 ± 0.05	0.574 ± 0.07	2.563 ± 0.07	2.004 ± 0.07	39.22 ± 4.25	22.54 ± 2.75
3	1.918 ± 0.12	1.862 ± 0.22	1.151 ± 0.47	0.494 ± 0.06	2.110 ± 0.01	0.876 ± 0.01	35.12 ± 0.94	36.29 ± 2.95
4	2.241 ± 0.26	1.604 ± 0.01	1.799 ± 0.10	0.562 ± 0.11	1.541 ± 0.18	1.741 ± 0.04	53.27 ± 3.61	24.32 ± 4.17
5	2.393 ± 0.30	1.788 ± 0.18	1.338 ± 0.05	0.545 ± 0.03	2.252 ± 0.08	1.487 ± 0.02	37.48 ± 0.59	26.75 ± 1.48
6	2.772 ± 0.17	1.746 ± 0.17	2.086 ± 0.09	0.753 ± 0.03	4.477 ± 0.90	1.487 ± 0.02	32.42 ± 2.02	33.58 ± 0.57
7	2.919 ± 0.05	2.136 ± 0.03	1.300 ± 0.05	0.715 ± 0.03	1.901 ± 0.06	1.031 ± 0.07	44.30 ± 0.74	41.29 ± 0.99
8	2.289 ± 0.09	2.082 ± 0.10	1.822 ± 0.19	0.940 ± 0.01	2.992 ± 0.01	0.876 ± 0.03	38.02 ± 1.00	52.26 ± 0.71
9	2.132 ± 0.11	1.807 ± 0.02	2.427 ± 0.02	0.957 ± 0.06	3.094 ± 0.07	1.345 ± 0.07	44.45 ± 0.47	41.31 ± 0.24
10	2.194 ± 0.12	1.945 ± 0.08	1.655 ± 0.17	0.668 ± 0.06	3.542 ± 0.42	1.746 ± 0.15	33.53 ± 2.08	27.92 ± 3.46
11	1.905 ± 0.02	1.593 ± 0.29	1.607 ± 0.03	0.744 ± 0.14	4.341 ± 0.20	1.186 ± 0.07	29.64 ± 0.85	38.44 ± 2.62
12	1.715 ± 0.13	1.705 ± 0.18	1.070 ± 0.04	0.362 ± 0.06	2.982 ± 0.02	1.367 ± 0.03	27.71 ± 1.25	19.59 ± 2.64
13	2.018 ± 0.13	1.750 ± 0.07	0.676 ± 0.02	0.294 ± 0.05	2.344 ± 0.03	1.229 ± 0.18	22.41 ± 0.90	19.59 ± 4.28
14	2.170 ± 0.04	1.830 ± 0.15	1.631 ± 0.06	0.447 ± 0.03	2.651 ± 0.06	1.414 ± 0.25	38.63 ± 1.17	24.20 ± 1.91
15	2.488 ± 0.17	2.109 ± 0.36	2.542 ± 0.05	0.727 ± 0.07	3.917 ± 0.02	1.888 ± 0.17	39.02 ± 0.31	27.90 ± 0.21
16	3.256 ± 0.09	3.011 ± 0.50	3.328 ± 0.06	0.872 ± 0.05	4.312 ± 0.56	2.629 ± 0.16	43.86 ± 1.48	25.31 ± 0.07
17	2.469 ± 0.22	1.914 ± 0.4	2.096 ± 0.20	0.668 ± 0.06	2.914 ± 0.04	1.474 ± 0.35	41.90 ± 1.10	32.03 ± 7.04
18	2.040 ± 0.14	1.990 ± 0.06	1.664 ± 0.03	0.613 ± 0.08	2.529 ± 0.12	1.819 ± 0.14	39.87 ± 0.08	25.35 ± 0.99
19	2.279 ± 0.27	1.715 ± 0.26	1.727 ± 0.10	0.817 ± 0.03	3.177 ± 0.05	1.681 ± 0.09	35.19 ± 0.91	32.69 ± 1.86
20	2.319 ± 0.24	1.762 ± 0.09	1.645 ± 0.08	0.740 ± 0.01	1.808 ± 0.45	1.651 ± 0.14	42.05 ± 0.74	31.07 ± 1.95
21	1.980 ± 0.07	1.933 ± 0.23	1.918 ± 0.13	0.672 ± 0.12	3.075 ± 0.01	1.729 ± 0.05	38.53 ± 0.19	27.92 ± 2.87
22	1.685 ± 0.03	1.530 ± 0.06	1.429 ± 0.15	0.532 ± 0.02	2.422 ± 0.01	2.276 ± 0.24	38.40 ± 0.62	19.64 ± 1.97
23	2.341 ± 0.14	1.876 ± 0.07	1.285 ± 0.01	0.405 ± 0.11	1.857 ± 0.12	1.431 ± 0.39	40.82 ± 0.35	21.68 ± 0.65
24	2.002 ± 0.02	1.715 ± 0.02	1.012 ± 0.09	0.298 ± 0.03	3.328 ± 0.09	1.431 ± 0.12	23.48 ± 0.38	16.65 ± 2.31
25	1.757 ± 0.05	1.681 ± 0.07	1.309 ± 0.29	0.375 ± 0.09	4.277 ± 0.23	1.690 ± 0.08	24.41 ± 0.61	18.07 ± 4.48
26	2.464 ± 0.17	1.811 ± 0.18	1.559 ± 0.01	0.613 ± 0.07	1.789 ± 0.03	1.324 ± 0.13	47.18 ± 2.26	31.64 ± 0.14
27	1.928 ± 0.06	1.903 ± 0.14	1.808 ± 0.17	0.570 ± 0.07	2.807 ± 0.15	1.242 ± 0.08	41.42 ± 0.96	31.48 ± 4.16
28	2.293 ± 0.01	1.864 ± 0.63	1.381 ± 0.04	0.778 ± 0.02	2.500 ± 0.07	1.716 ± 0.01	42.94 ± 2.16	31.28 ± 0.47
29	2.250 ± 0.02	2.175 ± 0.03	1.899 ± 0.18	1.152 ± 0.07	2.685 ± 0.19	1.302 ± 0.22	43.62 ± 1.73	46.84 ± 2.90
30	2.412 ± 0.08	2.296 ± 0.10	1.683 ± 0.01	0.778 ± 0.14	3.182 ± 0.09	1.487 ± 0.22	40.94 ± 0.73	34.22 ± 0.57
31	2.469 ± 0.02	1.708 ± 0.08	1.333 ± 0.05	0.867 ± 0.09	2.519 ± 0.13	1.332 ± 0.03	34.57 ± 1.05	39.31 ± 2.83
32	2.118 ± 0.01	1.925 ± 0.09	1.338 ± 0.04	0.715 ± 0.09	2.393 ± 0.06	1.642 ± 0.34	37.47 ± 0.49	30.53 ± 1.83
33	2.204 ± 0.06	1.729 ± 0.05	1.338 ± 0.13	0.778 ± 0.03	2.787 ± 0.11	1.229 ± 0.16	33.29 ± 0.51	39.06 ± 2.43
34	1.838 ± 0.04	1.906 ± 0.07	1.185 ± 0.01	0.693 ± 0.16	3.834 ± 0.04	1.733 ± 0.06	24.50 ± 1.78	28.49 ± 5.21
35	1.995 ± 0.03	1.922 ± 0.08	1.281 ± 0.08	0.681 ± 0.01	2.997 ± 0.09	1.922 ± 0.11	31.08 ± 0.35	26.37 ± 0.87
36	1.938 ± 0.06	1.692 ± 0.19	1.535 ± 0.04	0.642 ± 0.02	3.571 ± 0.09	1.965 ± 0.11	30.29 ± 0.40	24.91 ± 1.51
37	2.516 ± 0.03	2.422 ± 0.47	1.530 ± 0.06	0.727 ± 0.01	1.867 ± 0.02	1.113 ± 0.25	45.29 ± 1.42	40.51 ± 6.31
38	2.175 ± 0.03	2.143 ± 0.01	1.257 ± 0.02	0.719 ± 0.18	1.979 ± 0.01	1.418 ± 0.13	39.34 ± 0.67	33.55 ± 7.75
39	2.336 ± 0.20	1.841 ± 0.11	1.391 ± 0.03	0.409 ± 0.08	2.880 ± 0.04	1.087 ± 0.01	34.16 ± 2.54	26.72 ± 3.83
40	2.744 ± 0.02	2.273 ± 0.22	2.000 ± 0.01	0.413 ± 0.02	3.303 ± 0.01	1.836 ± 0.25	37.89 ± 3.56	18.75 ± 2.44
41	2.426 ± 0.03	2.033 ± 0.08	2.057 ± 0.09	0.676 ± 0.02	3.513 ± 0.20	1.237 ± 0.08	37.39 ± 1.04	35.52 ± 0.86
42	2.232 ± 0.01	1.757 ± 0.13	1.199 ± 0.08	0.600 ± 0.14	1.989 ± 0.14	1.319 ± 0.09	37.93 ± 0.39	30.78 ± 3.79
43	3.337 ± 0.11	1.845 ± 0.21	2.019 ± 0.02	0.693 ± 0.01	2.631 ± 0.06	0.979 ± 0.03	43.43 ± 1.54	41.92 ± 0.44
44	2.156 ± 0.05	1.528 ± 0.07	0.782 ± 0.01	0.222 ± 0.04	3.372 ± 0.07	1.113 ± 0.04	18.86 ± 0.86	15.00 ± 2.99
45	2.806 ± 0.01	2.151 ± 0.19	2.101 ± 0.07	0.413 ± 0.19	3.211 ± 0.05	1.207 ± 0.07	40.36 ± 0.30	23.91 ± 8.59
46	2.720 ± 0.01	1.830 ± 0.17	1.242 ± 0.01	0.358 ± 0.02	4.901 ± 0.04	1.914 ± 0.07	20.02 ± 1.11	15.95 ± 1.07
47	2.086 ± 0.03	1.667 ± 0.07	0.945 ± 0.04	0.315 ± 0.03	1.955 ± 0.08	1.255 ± 0.16	33.09 ± 0.10	19.39 ± 0.47
48	2.910 ± 0.06	2.621 ± 0.27	1.372 ± 0.09	0.965 ± 0.02	4.073 ± 0.06	3.137 ± 0.06	22.39 ± 0.74	24.18 ± 0.06
49	3.147 ± 0.07	1.796 ± 0.42	2.105 ± 0.19	0.685 ± 0.02	2.812 ± 0.05	1.466 ± 0.09	42.89 ± 0.61	31.91 ± 2.01
50	2.235 ± 0.01	2.132 ± 0.10	1.635 ± 0.06	0.651 ± 0.11	2.291 ± 0.10	1.595 ± 0.37	42.03 ± 0.62	29.92 ± 8.38
51	2.075 ± 0.03	1.861 ± 0.31	1.717 ± 0.10	0.553 ± 0.14	3.196 ± 0.08	1.255 ± 0.17	35.74 ± 1.24	30.56 ± 8.36
52	2.991 ± 0.04	2.736 ± 0.47	2.120 ± 0.04	0.757 ± 0.20	4.993 ± 0.02	0.828 ± 0.14	30.49 ± 0.32	48.28 ± 11.33
53	2.606 ± 0.16	2.105 ± 0.08	1.933 ± 0.03	0.935 ± 0.07	4.701 ± 0.11	1.147 ± 0.26	29.37 ± 1.10	45.48 ± 8.03
54	2.493 ± 0.15	2.334 ± 0.07	1.592 ± 0.03	1.241 ± 0.02	3.849 ± 0.05	1.281 ± 0.14	29.86 ± 0.19	48.77 ± 3.16
55	1.738 ± 0.39	1.710 ± 0.07	1.003 ± 0.12	0.477 ± 0.04	2.446 ± 0.02	1.380 ± 0.12	29.31 ± 0.54	25.61 ± 3.13
56	2.483 ± 0.07	1.937 ± 0.36	1.688 ± 0.06	0.583 ± 0.02	2.739 ± 0.04	1.828 ± 0.43	40.11 ± 0.62	25.13 ± 4.90
57	2.445 ± 0.05	1.849 ± 0.21	1.424 ± 0.08	0.252 ± 0.05	3.021 ± 0.02	2.642 ± 0.64	32.03 ± 0.25	10.25 ± 3.04
58	2.506 ± 0.09	2.066 ± 0.05	2.206 ± 0.03	1.369 ± 0.58	3.834 ± 0.12	1.772 ± 0.17	36.94 ± 0.90	41.62 ± 12.48
59	2.853 ± 0.07	2.625 ± 0.14	3.568 ± 0.15	1.207 ± 0.03	3.888 ± 0.02	1.681 ± 0.18	48.04 ± 0.51	41.40 ± 3.14
60	2.203 ± 0.11	1.468 ± 0.02	1.583 ± 0.07	0.613 ± 0.20	3.698 ± 0.11	1.660 ± 0.10	35.72 ± 4.05	26.43 ± 5.22
Heritability	0.95	0.76	0.96	0.85	0.97	0.86	0.97	0.85
Mean	2.33	1.95	1.65	0.66	3.06	1.53	35.99	30.12
Range (mean)	1.69–3.34	1.53–3.01	0.68–3.57	0.22–1.37	1.54–4.99	0.83–3.14	18.86–53.27	52.26–10.25
SE±	0.086	0.160	0.077	0.073	0.108	0.140	1.084	3.152
Range (SE±)	0.02–0.30	0.01–0.63	0.01–0.47	0.01–0.58	0.01–0.90	0.00–0.64	0.08–4.25	0.07–12.48
LSD (0.05)	0.20 **	0.34 **	0.21 **	0.20 **	0.29 **	0.34 **	2.77 **	7.85 **
CV (%)	6.38	13.72	9.32	23.63	6.69	17.04	5.53	20.02

** *p* ≤ 0.001; ns., not significant; OS, optimum sowing and LS, late sowing; HSW, 100-seed weight; GYP, grain yield/plant; SYP, straw yield/plant and HI, harvest index.

**Table 3:** Ranks (R), Ranks mean (MR) and standard deviation of ranks (SDR) of several heat-tolerance indicators (Yp, Ys, TOL, SSI, RHI, MP, STI, GMP and YI) of 60 lentil genotypes.

Genotype	Yp	R	Ys	R	TOL	R	SSI	R	RHI	R	MP	R	STI	R	GMP	R	YI	R
1	1.62	30	0.46	46	1.16	41	1.19	48	0.71	48	1.04	39	0.28	43	0.86	43	0.70	46
2	1.86	17	0.56	39	1.30	48	1.16	44	0.76	44	1.21	20	0.38	28	1.02	28	0.85	39
3	1.13	54	0.46	45	0.66	16	0.98	23	1.03	23	0.80	53	0.19	49	0.72	49	0.71	45
4	1.81	20	0.54	41	1.26	46	1.16	45	0.75	45	1.17	26	0.36	31	0.99	31	0.83	41
5	1.33	42	0.52	43	0.80	22	1.01	27	0.99	27	0.93	47	0.26	46	0.83	46	0.80	43
6	2.11	10	0.77	16	1.34	50	1.05	30	0.92	30	1.44	10	0.60	11	1.27	11	1.17	16
7	1.29	47	0.73	22	0.56	9	0.72	10	1.42	10	1.01	42	0.34	37	0.97	37	1.11	22
8	1.83	18	0.99	7	0.84	23	0.76	13	1.36	13	1.41	11	0.67	9	1.35	9	1.51	7
9	2.46	4	1.01	6	1.45	55	0.98	24	1.03	24	1.73	4	0.91	4	1.57	4	1.53	6
10	1.65	26	0.67	30	0.98	36	0.99	25	1.02	25	1.16	27	0.41	24	1.05	24	1.02	30
11	1.61	31	0.76	17	0.84	24	0.87	18	1.19	18	1.18	25	0.45	21	1.11	21	1.16	17
12	1.05	55	0.31	54	0.73	18	1.17	46	0.75	46	0.68	56	0.12	55	0.57	55	0.48	54
13	0.63	60	0.23	58	0.40	3	1.06	32	0.91	32	0.43	60	0.05	59	0.38	59	0.35	58
14	1.63	29	0.41	48	1.22	43	1.24	53	0.64	53	1.02	41	0.25	48	0.82	48	0.63	48
15	2.58	3	0.74	20	1.84	58	1.19	47	0.72	47	1.66	5	0.70	8	1.38	8	1.13	20
16	3.40	2	0.91	9	2.49	60	1.22	51	0.67	51	2.15	2	1.14	3	1.76	3	1.38	9
17	2.11	9	0.67	31	1.44	54	1.13	41	0.80	41	1.39	13	0.52	15	1.19	15	1.02	31
18	1.67	25	0.61	34	1.06	39	1.06	31	0.92	31	1.14	29	0.37	29	1.01	29	0.93	34
19	1.73	21	0.84	11	0.89	26	0.85	16	1.22	16	1.29	17	0.54	13	1.21	13	1.29	11
20	1.65	27	0.76	18	0.89	28	0.90	20	1.15	20	1.20	21	0.46	20	1.11	20	1.15	18
21	1.93	15	0.67	29	1.26	45	1.08	36	0.88	36	1.30	16	0.48	19	1.14	19	1.03	29
22	1.42	37	0.51	44	0.91	29	1.06	34	0.91	34	0.96	45	0.27	45	0.85	45	0.78	44
23	1.27	48	0.36	52	0.91	30	1.19	49	0.71	49	0.82	50	0.17	51	0.68	51	0.55	52
24	0.99	56	0.23	57	0.75	20	1.27	56	0.59	56	0.61	57	0.08	58	0.48	58	0.35	57
25	1.30	46	0.32	53	0.97	34	1.25	54	0.63	54	0.81	51	0.16	53	0.65	53	0.50	53
26	1.56	34	0.60	35	0.95	32	1.02	28	0.97	28	1.08	37	0.35	36	0.97	36	0.92	35
27	1.82	19	0.55	40	1.26	47	1.16	43	0.77	43	1.19	24	0.37	30	1.00	30	0.85	40
28	1.37	40	0.80	12	0.57	10	0.69	9	1.47	9	1.09	35	0.41	25	1.05	25	1.22	12
29	1.91	16	1.24	4	0.67	17	0.58	5	1.63	5	1.58	6	0.88	5	1.54	5	1.90	4
30	1.69	24	0.80	14	0.89	25	0.87	19	1.19	19	1.24	19	0.50	18	1.16	18	1.22	14
31	1.32	45	0.91	10	0.41	4	0.52	3	1.72	3	1.11	33	0.44	22	1.09	22	1.38	10
32	1.33	43	0.72	23	0.60	14	0.76	11	1.37	11	1.02	40	0.35	34	0.98	34	1.10	23
33	1.33	44	0.80	13	0.53	7	0.66	6	1.52	6	1.06	38	0.39	27	1.03	27	1.22	13
34	1.17	53	0.70	25	0.47	5	0.66	7	1.51	7	0.93	46	0.30	41	0.90	41	1.07	25
35	1.27	49	0.69	27	0.58	11	0.76	12	1.36	12	0.98	44	0.32	40	0.93	40	1.04	27
36	1.53	35	0.64	33	0.89	27	0.97	22	1.05	22	1.08	36	0.36	32	0.99	32	0.98	33
37	1.53	36	0.74	19	0.78	21	0.85	17	1.22	17	1.13	30	0.42	23	1.06	23	1.13	19
38	1.24	50	0.73	21	0.51	6	0.68	8	1.48	8	0.99	43	0.34	39	0.95	39	1.12	21
39	1.38	39	0.36	51	1.02	38	1.22	52	0.66	52	0.87	49	0.18	50	0.71	50	0.55	51
40	2.02	13	0.37	49	1.64	56	1.36	58	0.46	58	1.19	22	0.28	44	0.86	44	0.57	49
41	2.08	11	0.68	28	1.39	52	1.12	39	0.83	39	1.38	14	0.52	16	1.19	16	1.04	28
42	1.18	52	0.59	37	0.60	12	0.84	15	1.25	15	0.88	48	0.26	47	0.83	47	0.89	37
43	2.03	12	0.70	24	1.33	49	1.09	37	0.87	37	1.37	15	0.53	14	1.19	14	1.07	24
44	0.75	59	0.15	60	0.60	13	1.34	57	0.49	57	0.45	59	0.04	60	0.33	60	0.22	60
45	2.12	8	0.37	50	1.75	57	1.37	59	0.44	59	1.25	18	0.29	42	0.89	42	0.56	50
46	1.23	51	0.30	55	0.92	31	1.25	55	0.62	55	0.76	54	0.14	54	0.61	54	0.46	55
47	0.91	58	0.26	56	0.66	15	1.20	50	0.70	50	0.58	58	0.09	57	0.48	57	0.39	56
48	1.36	41	1.02	5	0.34	2	0.42	2	1.88	2	1.19	23	0.51	17	1.18	17	1.55	5
49	2.13	7	0.69	26	1.44	53	1.12	40	0.81	40	1.41	12	0.54	12	1.21	12	1.05	26
50	1.64	28	0.65	32	0.98	37	1.00	26	1.00	26	1.14	28	0.39	26	1.03	26	0.99	32
51	1.72	22	0.53	42	1.19	42	1.15	42	0.78	42	1.13	32	0.34	38	0.96	38	0.81	42
52	2.14	6	0.77	15	1.36	51	1.06	33	0.91	33	1.46	9	0.61	10	1.29	10	1.18	15
53	1.95	14	0.98	8	0.96	33	0.82	14	1.27	14	1.46	8	0.70	7	1.38	7	1.50	8
54	1.59	32	1.35	2	0.24	1	0.26	1	2.12	1	1.47	7	0.79	6	1.46	6	2.05	2
55	0.98	57	0.44	47	0.53	8	0.91	21	1.14	21	0.71	55	0.16	52	0.66	52	0.68	47
56	1.69	23	0.57	38	1.12	40	1.10	38	0.85	38	1.13	31	0.36	33	0.98	33	0.87	38
57	1.41	38	0.18	59	1.23	44	1.45	60	0.32	60	0.80	52	0.09	56	0.51	56	0.28	59
58	2.23	5	1.49	1	0.73	19	0.55	4	1.68	4	1.86	3	1.23	2	1.82	2	2.28	1
59	3.65	1	1.30	3	2.35	59	1.07	35	0.90	35	2.48	1	1.76	1	2.18	1	1.99	3
60	1.58	33	0.60	36	0.97	35	1.03	29	0.96	29	1.09	34	0.35	35	0.98	35	0.92	36

Note: Stress-susceptibility index (SSI), relative heat index (RHI), tolerance (TOL), mean productivity (MP), stress-tolerance index (STI), geometric mean productivity (GMP), yield index (YI), yield-stability index (YSI), heat-resistance index (HRI), modified stress-tolerance index (MSTI), abiotic tolerance index (ATI) and stress-susceptibility percentage (SSPI).

**Table 4:** Ranks (R), Ranks mean (MR) and standard deviation of ranks (SDR) of several heat-tolerance indicators (YSI, HRI, ATI, SSPI, K_1_STI and K_2_STI) of 60 lentil genotypes.

Genotype	YSI	R	HRI	R	ATI	R	SSPI	R	K_1_STI	R	K_2_STI	R	MR	SDR	RS
1	0.28	48	0.20	47	0.40	33	35.25	41	0.27	38	0.14	45	42.40	5.33	47.73
2	0.30	44	0.26	44	0.53	45	39.40	48	0.49	19	0.28	37	36.27	10.57	46.84
3	0.41	23	0.29	41	0.19	11	20.17	16	0.09	52	0.10	47	36.47	15.15	51.62
4	0.30	45	0.25	46	0.50	43	38.38	46	0.44	23	0.25	40	37.93	8.86	46.79
5	0.40	27	0.32	38	0.27	23	24.36	22	0.17	46	0.16	43	36.13	9.70	45.83
6	0.37	30	0.43	23	0.68	53	40.54	50	0.98	9	0.82	14	24.20	15.24	39.44
7	0.56	10	0.62	15	0.22	15	17.00	9	0.21	42	0.42	28	23.67	13.51	37.18
8	0.54	13	0.82	6	0.45	41	25.46	23	0.82	13	1.52	8	14.27	8.82	23.09
9	0.41	24	0.63	14	0.91	57	44.16	55	2.04	4	2.15	6	19.40	19.61	39.01
10	0.41	25	0.42	25	0.41	37	29.83	36	0.41	25	0.43	26	28.07	4.49	32.56
11	0.47	18	0.55	19	0.37	29	25.61	24	0.43	24	0.61	16	21.47	4.41	25.88
12	0.30	46	0.14	51	0.17	8	22.27	18	0.05	56	0.03	55	44.87	15.64	60.51
13	0.36	32	0.13	52	0.06	1	12.26	3	0.01	60	0.01	59	41.87	22.32	64.19
14	0.25	53	0.16	48	0.40	32	36.96	43	0.24	40	0.10	46	44.87	6.89	51.75
15	0.29	47	0.32	37	1.01	58	55.92	58	1.73	5	0.89	11	28.80	21.22	50.02
16	0.27	51	0.37	29	1.75	59	75.71	60	4.85	2	2.18	5	26.40	24.61	51.01
17	0.32	41	0.33	36	0.68	54	43.73	54	0.86	11	0.55	23	31.27	15.69	46.96
18	0.36	31	0.34	35	0.42	38	32.11	39	0.38	28	0.32	34	32.40	4.06	36.46
19	0.49	16	0.63	13	0.43	39	26.90	26	0.59	17	0.89	10	17.67	7.43	25.09
20	0.46	20	0.53	21	0.40	31	27.02	28	0.46	21	0.61	17	22.00	4.15	26.15
21	0.35	36	0.36	30	0.57	49	38.11	45	0.66	16	0.51	25	29.67	10.96	40.63
22	0.36	34	0.28	42	0.31	25	27.51	29	0.20	43	0.16	44	38.27	6.71	44.97
23	0.28	49	0.16	49	0.25	20	27.62	30	0.10	50	0.05	52	45.47	9.71	55.17
24	0.24	56	0.08	58	0.14	6	22.88	20	0.03	57	0.01	57	48.60	16.91	65.51
25	0.25	54	0.12	53	0.25	22	29.46	34	0.10	51	0.04	53	47.87	9.48	57.35
26	0.39	28	0.36	31	0.37	28	28.95	32	0.31	34	0.30	36	32.67	3.24	35.91
27	0.30	43	0.26	43	0.51	44	38.40	47	0.45	22	0.27	39	36.93	9.06	45.99
28	0.59	9	0.72	9	0.24	19	17.22	10	0.28	37	0.61	18	18.60	10.81	29.41
29	0.65	5	1.23	3	0.41	35	20.24	17	1.18	6	3.15	4	9.13	8.41	17.55
30	0.47	19	0.58	17	0.41	36	26.89	25	0.52	18	0.74	15	20.00	5.44	25.44
31	0.69	3	0.95	5	0.18	10	12.58	4	0.28	36	0.84	13	14.87	13.21	28.07
32	0.54	11	0.60	16	0.24	18	18.34	14	0.23	41	0.43	27	24.00	11.33	35.33
33	0.60	6	0.74	8	0.22	16	15.94	7	0.25	39	0.58	21	18.53	12.94	31.47
34	0.60	7	0.64	12	0.17	9	14.13	5	0.15	47	0.34	33	24.20	17.27	41.47
35	0.54	12	0.57	18	0.22	14	17.61	11	0.19	45	0.35	31	26.20	13.85	40.05
36	0.42	22	0.41	26	0.35	27	26.99	27	0.31	35	0.34	32	29.40	4.80	34.20
37	0.49	17	0.55	20	0.33	26	23.79	21	0.36	31	0.53	24	22.93	5.47	28.40
38	0.59	8	0.66	11	0.19	12	15.54	6	0.19	44	0.42	29	23.00	15.59	38.59
39	0.26	52	0.15	50	0.29	24	30.82	38	0.13	49	0.06	51	46.40	7.78	54.18
40	0.18	58	0.10	56	0.57	48	49.93	56	0.41	26	0.09	49	45.73	13.75	59.48
41	0.33	39	0.34	33	0.66	52	42.28	52	0.83	12	0.57	22	30.20	14.40	44.60
42	0.50	15	0.44	22	0.20	13	18.07	12	0.13	48	0.21	42	30.80	15.53	46.33
43	0.35	37	0.37	28	0.63	51	40.43	49	0.80	14	0.60	19	28.27	13.61	41.88
44	0.20	57	0.04	59	0.08	2	18.19	13	0.01	59	0.00	60	49.00	20.00	69.00
45	0.17	59	0.10	57	0.62	50	53.13	57	0.48	20	0.09	48	45.07	16.01	61.08
46	0.25	55	0.11	54	0.22	17	27.97	31	0.08	53	0.03	54	48.53	11.53	60.06
47	0.28	50	0.11	55	0.13	3	20.03	15	0.03	58	0.01	56	46.27	18.02	64.29
48	0.75	2	1.17	4	0.16	7	10.32	2	0.35	32	1.24	9	11.33	11.84	23.17
49	0.32	40	0.34	34	0.69	55	43.61	53	0.90	10	0.60	20	29.33	16.38	45.71
50	0.40	26	0.40	27	0.40	34	29.87	37	0.39	27	0.39	30	29.47	3.86	33.33
51	0.31	42	0.25	45	0.45	42	36.08	42	0.37	30	0.22	41	38.80	5.96	44.76
52	0.36	33	0.43	24	0.70	56	41.43	51	1.03	7	0.85	12	24.33	16.85	41.18
53	0.50	14	0.76	7	0.53	46	29.25	33	0.98	8	1.58	7	15.20	11.73	26.93
54	0.85	1	1.73	1	0.14	5	7.43	1	0.74	15	3.32	3	5.60	7.95	13.55
55	0.46	21	0.31	39	0.14	4	16.13	8	0.06	55	0.07	50	35.80	18.95	54.75
56	0.34	38	0.30	40	0.44	40	33.99	40	0.38	29	0.27	38	35.80	4.82	40.62
57	0.13	60	0.04	60	0.25	21	37.38	44	0.07	54	0.01	58	52.07	10.68	62.75
58	0.67	4	1.53	2	0.53	47	22.31	19	2.25	3	6.37	2	7.87	11.87	19.73
59	0.36	35	0.71	10	2.04	60	71.28	59	8.65	1	6.95	1	20.33	23.32	43.66
60	0.38	29	0.35	32	0.38	30	29.60	35	0.32	33	0.30	35	33.07	2.54	35.61

Note: stress-susceptibility index (SSI), relative heat index (RHI), tolerance (TOL), mean productivity (MP), stress-tolerance index (STI), geometric mean productivity (GMP), yield index (YI), yield-stability index (YSI), heat-resistance index (HRI), modified stress-tolerance index (modified stress-tolerance index), abiotic tolerance index (ATI) and stress-susceptibility percentage (SSPI).

**Table 5:** Correlation coefficients of yield with stress indices under stress and non-stress conditions in 60 lentil genotypes.

Indices	Yp	Ys	TOL	SSI	RHI	MP	STI	GMP	YI	YSI	HRI	ATI	SSPI	K1STI	k2STI
Yp	1.00	0.55 **	0.85 **	0.12 ^ns^	−0.12 ^ns^	0.95 **	0.85 **	0.85 **	0.55 **	0.12 ^ns^	0.19 ^ns^	0.95 **	0.85 **	0.82 **	0.62 **
Ys		1.00	0.03 ^ns^	−0.74 **	0.74 **	0.79 **	0.86 **	0.91 **	0.97 **	0.74 **	0.90 **	0.41 **	0.03 ^ns^	0.54 **	0.81 **
TOL			1.00	0.61 **	−0.61**	0.64 **	0.47 **	0.44 **	0.03 ^ns^	−0.61 **	−0.35 *	0.89 **	0.99 **	0.65 **	0.23 ^ns^
SSI				1.00	−0.98 **	−0.20 ^ns^	−0.33 *	−0.41 **	−0.74 **	−0.99 **	−0.91 *	0.24 ^ns^	0.61 **	0.01 ^ns^	−0.40 **
RHI					1.00	0.20 ^ns^	0.33 *	0.41 **	0.74 **	0.99 **	0.91 **	−0.24 ^ns^	−0.61 **	−0.01 ^ns^	0.40 **
MP						1.00	0.95 **	0.97 **	0.79 **	0.20 ^ns^	0.49 **	0.86 **	0.64 **	0.81**	0.77 **
STI							1.00	0.97 **	0.86 **	0.33 *	0.61 **	0.79 **	0.47 **	0.87 **	0.91 **
GMP								1.00	0.91 **	0.41 **	0.65 **	0.74 **	0.44 **	0.75 **	0.81 **
YI									1.00	0.74 **	0.90 **	0.41 **	0.03 ^ns^	0.54 **	0.81 **
YSI										1.00	0.91 **	−0.24 ^ns^	−0.61 **	−0.01 ^ns^	0.4 **
HRI											1.00	0.02 ^ns^	−0.35 *	0.24 ^ns^	0.68 **
ATI												1.00	0.89 **	0.90 **	0.57 **
SSPI													1.00	0.65 **	0.23 ^ns^
K_1_STI														1.00	0.79 **
K_2_STI															1.00

Note: stress-susceptibility index (SSI), relative heat index (RHI), tolerance (TOL), mean productivity (MP), stress-tolerance index (STI), geometric mean productivity (GMP), yield index (YI), yield-stability index (YSI), heat-resistance index (HRI), modified stress-tolerance index (MSTI), abiotic tolerance index (ATI) and stress-susceptibility percentage (SSPI). * *p* ≤ 0.05, ** *p* ≤ 0.001 and ns., not significant

**Table 6:** Different thermal unit indices of 60 lentil genotypes with their days to maturity (DM) and yield performance (kg ha^−1^).

Genotypes	DM		Yield kg ha^−1^		GDD		Duration of Sunshine Hours		HTU		PTI		HUE	
	OS	LS	OS	LS	OS	LS	OS	LS	OS	LS	OS	LS	OS	LS
1	114	97	3307	909	77.60	86.60	6.39	5.98	495.86	517.81	0.68	0.89	42.62	10.50
2	119	99	4316	1625	101.59	87.00	6.37	6.03	647.14	524.31	0.85	0.88	42.49	18.69
3	113	97	3291	1226	77.57	86.60	6.36	5.98	493.35	517.81	0.69	0.89	42.42	14.16
4	113	100	3291	1822	77.57	87.20	6.36	6.06	493.35	528.19	0.69	0.87	42.42	20.90
5	113	96	3291	1532	77.57	86.50	6.36	6.03	493.35	521.29	0.69	0.90	42.42	17.72
6	114	97	3307	1801	77.60	86.60	6.39	5.98	495.86	517.81	0.68	0.89	42.62	20.79
7	93	86	3204	1693	77.36	83.70	6.21	5.93	480.43	496.22	0.83	0.97	41.42	20.23
8	110	94	3275	2182	77.21	85.80	6.36	6.01	491.06	515.48	0.70	0.91	42.42	25.44
9	110	92	3275	2180	77.21	85.20	6.36	5.99	491.06	510.23	0.70	0.93	42.42	25.59
10	114	97	3307	1773	77.59	86.60	6.39	5.98	495.80	517.81	0.68	0.89	42.62	20.48
11	114	98	3307	1896	77.59	86.70	6.39	6.01	495.80	521.01	0.68	0.88	42.62	21.87
12	111	97	3278	1173	77.39	86.60	6.35	5.98	491.43	517.81	0.70	0.89	42.35	13.55
13	112	96	3356	1087	79.49	86.50	6.33	6.03	503.17	521.29	0.71	0.90	42.22	12.58
14	116	98	4344	1371	101.93	86.70	6.39	6.01	651.33	521.01	0.88	0.88	42.62	15.82
15	116	103	4344	1520	101.93	87.50	6.39	6.10	651.33	533.99	0.88	0.85	42.62	17.37
16	117	101	4351	1736	101.93	87.30	6.40	6.09	652.35	531.90	0.87	0.86	42.69	19.88
17	114	96	3307	1537	77.60	86.50	6.39	6.03	495.86	521.29	0.68	0.90	42.62	17.78
18	115	98	4371	1670	102.23	86.70	6.41	6.01	655.29	521.01	0.89	0.88	42.75	19.27
19	115	98	4371	1471	102.23	86.70	6.41	6.01	655.29	521.01	0.89	0.88	42.75	16.97
20	114	98	3307	1282	77.60	86.70	6.39	6.01	495.86	521.01	0.68	0.88	42.62	14.78
21	114	98	3307	1493	77.60	86.70	6.39	6.01	495.86	521.01	0.68	0.88	42.62	17.22
22	113	99	3291	1050	77.57	87.00	6.36	6.03	493.35	524.31	0.69	0.88	42.42	12.08
23	114	99	3307	1194	77.60	87.00	6.39	6.03	495.86	524.31	0.68	0.88	42.62	13.73
24	113	97	3291	989	77.57	86.60	6.36	5.98	493.35	517.81	0.69	0.89	42.42	11.42
25	113	98	3291	995	77.57	86.70	6.36	6.01	493.35	521.01	0.69	0.88	42.42	11.48
26	113	98	3291	1650	77.57	86.70	6.36	6.01	493.35	521.01	0.69	0.88	42.42	19.03
27	112	96	3356	1625	79.49	86.50	6.33	6.03	503.17	521.29	0.71	0.90	42.22	18.80
28	114	97	3307	2118	77.60	86.60	6.39	5.98	495.86	517.81	0.68	0.89	42.62	24.46
29	113	92	3291	2413	77.57	85.20	6.36	5.99	493.35	510.23	0.69	0.93	42.42	28.33
30	112	95	3356	2063	79.49	86.10	6.33	6.06	503.17	521.77	0.71	0.91	42.22	23.96
31	112	98	3356	2186	79.49	86.70	6.33	6.01	503.17	521.01	0.71	0.88	42.22	25.22
32	113	99	3291	1667	77.57	87.00	6.36	6.03	493.35	524.31	0.69	0.88	42.42	19.17
33	114	98	3307	2623	77.60	86.70	6.39	6.01	495.86	521.01	0.68	0.88	42.62	30.26
34	113	98	3291	2032	77.57	86.70	6.36	6.01	493.35	521.01	0.69	0.88	42.42	23.43
35	114	97	3307	1951	77.60	86.60	6.39	5.98	495.86	517.81	0.68	0.89	42.62	22.53
36	113	99	3291	1807	77.57	87.00	6.36	6.03	493.35	524.31	0.69	0.88	42.42	20.78
37	115	96	4371	1702	102.23	86.50	6.41	6.03	655.29	521.29	0.89	0.90	42.75	19.69
38	113	99	3291	1786	77.57	87.00	6.36	6.03	493.35	524.31	0.69	0.88	42.42	20.55
39	114	99	3307	1484	77.60	87.00	6.39	6.03	495.86	524.31	0.68	0.88	42.62	17.07
40	107	104	4406	920	101.93	87.70	6.48	6.11	660.51	535.66	0.95	0.84	43.22	10.49
41	114	103	3307	1653	77.60	87.50	6.39	6.10	495.86	533.99	0.68	0.85	42.62	18.88
42	114	97	3307	1721	77.60	86.60	6.39	5.98	495.86	517.81	0.68	0.89	42.62	19.87
43	113	94	3291	1851	77.57	85.80	6.36	6.01	493.35	515.48	0.69	0.91	42.42	21.58
44	114	94	3307	735	77.60	85.80	6.39	6.01	495.86	515.48	0.68	0.91	42.62	8.57
45	115	103	4371	752	102.23	87.50	6.41	6.10	655.29	533.99	0.89	0.85	42.75	8.59
46	117	106	4331	771	101.93	87.70	6.37	6.14	649.29	538.42	0.87	0.83	42.49	8.80
47	115	102	4371	680	102.23	87.40	6.41	6.09	655.29	532.45	0.89	0.86	42.75	7.77
48	112	107	3356	1870	79.49	87.70	6.33	6.15	503.17	539.11	0.71	0.82	42.22	21.33
49	113	98	3291	1940	77.57	86.70	6.36	6.01	493.35	521.01	0.69	0.88	42.42	22.37
50	114	99	3307	2017	77.60	87.00	6.39	6.03	495.86	524.31	0.68	0.88	42.62	23.20
51	115	98	4371	1931	102.23	86.70	6.41	6.01	655.29	521.01	0.89	0.88	42.75	22.27
52	110	91	3275	1878	77.21	84.90	6.36	5.95	491.06	504.86	0.70	0.93	42.42	22.14
53	109	91	3303	2158	77.14	84.90	6.42	5.95	495.24	504.86	0.71	0.93	42.82	25.43
54	109	90	3303	1784	77.14	76.70	6.42	5.90	495.24	452.53	0.71	0.85	42.82	23.26
55	113	98	3291	1140	77.57	86.70	6.36	6.01	493.35	521.01	0.69	0.88	42.42	13.15
56	114	105	3307	1363	77.60	87.50	6.39	6.12	495.86	535.74	0.68	0.83	42.62	15.57
57	115	104	4371	502	102.23	87.70	6.41	6.11	655.29	535.66	0.89	0.84	42.75	5.72
58	113	98	3291	2269	77.57	86.70	6.36	6.01	493.35	521.01	0.69	0.88	42.42	26.17
59	110	91	3275	2274	77.21	84.90	6.36	5.95	491.06	504.86	0.70	0.93	42.42	26.80
60	115	98	4371	1482	102.23	86.70	6.41	6.01	655.29	521.01	0.89	0.88	42.75	17.10

**Table 7:** Chlorophyll content with standard error of 10 screened lentil genotypes (eight HT and two HS) under OS and LS conditions during the 2020–2021 crop-growing season at PRC, BARI, Ishurdi, Pabna.

Genotypes	Chl a (mmol g^−1^)		Chl b (mmol g^−1^)		Total Chl (mmol g^−1^)	
	OS	LS	OS	LS	OS	LS
BLX 09015 (HT)	1.05 ± 0.09	0.93 ± 0.08	0.57 ± 0.18	0.24 ± 0.02	1.61 ± 0.09	1.17 ± 0.09
PRECOZ (HT)	1.17 ± 0.09	0.69 ± 0.11	0.6 ± 0.29	0.25 ± 0.04	1.77 ± 0.36	0.94 ± 0.15
BLX 05002-3 (HT)	1.24 ± 0.22	0.8 ± 0.10	0.49 ± 0.08	0.21 ± 0.01	1.73 ± 0.30	1.01 ± 0.11
LRL-21-112-1-1-1-1-6 (HT)	1.86 ± 0.23	1.09 ± 0.15	0.65 ± 0.23	0.35 ± 0.05	2.51 ± 0.41	1.44 ± 0.2
LR-9-25 (HT)	1.24 ± 0.13	0.94 ± 0.05	0.93 ± 0.32	0.41 ± 0.02	2.17 ± 0.41	1.35 ± 0.07
BLX 05002-6 (HT)	1.72 ± 0.12	1.1 ± 0.07	0.43 ± 0.06	0.39 ± 0.05	2.14 ± 0.16	1.49 ± 0.11
BARI Masur-8 (HT)	1.81 ± 0.41	1.18 ± 0.09	1.09 ± 0.18	0.38 ± 0.04	2.9 ± 0.59	1.56 ± 0.13
RL-12-181 (HT)	1.55 ± 0.11	1.14 ± 0.02	0.81 ± 0.28	0.28 ± 0.11	2.36 ± 0.35	1.42 ± 0.10
BLX 12009-6 (HS)	1.7 ± 0.20	1.05 ± 0.08	0.81 ± 0.22	0.41 ± 0.05	2.51 ± 0.15	1.46 ± 0.13
LG 198 (HS)	1.46 ± 0.27	0.89 ± 0.14	0.48 ± 0.14	0.32 ± 0.07	1.94 ± 0.36	1.21 ± 0.21
Heritability	0.55	0.133	1.51E-17	5.56E-15	0.31	0.054
Mean	1.479	0.981	0.69	0.324	2.17	1.305
Range (Mean)	1.05–1.86	0.80–1.18	0.43–1.09	0.21–0.41	1.61–2.90	0.94–1.56
SE	0.19	0.17	0.20	0.09	0.32	0.25
Range (SE)	0.09–0.41	0.02–0.15	0.06–0.29	0.01–0.11	0.09–0.59	0.07–0.21
LSD (0.05)	0.60 ^ns^	0.44 ^ns^	0.66 ^ns^	0.24 ^ns^	1.01 ^ns^	0.62 ^ns^
CV (%)	22.78	26.17	56.12	42.25	27.26	27.50

Values are mean ± SE. (*n* = 3), ^ns^ Not significant, HT is heat tolerant, HS is heat susceptible.

**Table 8:** Mean value of proline and relative water content (RWC) with increasing or reduction percentage of 10 selective genotypes under optimum- and late-sowing conditions 2020–2021 at PRC, Ishurdi, Pabna.

Genotypes	Proline Content (mg g^−1^)			Relative Water Content (%)		
	OS	LS	Increasing (%)	OS	LS	Reduction (%)
BLX 09015 (HT)	0.66 ± 0.022	0.91 ± 0.01	3.749	83.67 ± 1.867	77.67 ± 0.5	7.17
PRECOZ (HT)	0.43 ± 0.013	0.7 ± 0.00	6.28	80.67 ± 2.515	73.67 ± 1.98	8.68
BLX 05002-3 (HT)	0.49 ± 0.003	0.92 ± 0.01	8.78	81.00 ± 2.046	73.33 ± 2.36	9.47
LRL-21-112-1-1-1-1-6 (HT)	0.47 ± 0.003	0.65 ± 0.1	3.83	80.33 ± 1.720	73.67 ± 10.86	8.29
LR-9-25 (HT)	0.8 ± 0.054	1.35 ± 0.04	6.88	79.67 ± 0.840	73.67 ± 0.51	7.53
BLX 05002-6 (HT)	0.68 ± 0.004	0.95 ± 0.02	3.97	80.00 ± 2.707	74 ± 1.74	7.50
BARI Masur-8 (HT)	0.63 ± 0.003	0.82 ± 0.02	3.02	76.67 ± 3.021	70.67 ± 1.71	7.83
RL-12-181 (HT)	0.53 ± 0.037	0.84 ± 0.01	5.85	82.33 ± 0.984	69.67 ± 0.84	15.38
BLX 12009-6 (HS)	0.46 ± 0.023	0.63 ± 0.00	3.70	80.00 ± 1.443	61.67 ± 1.07	22.91
LG-198 (HS)	0.71 ± 0.075	0.75 ± 0.01	0.56	83.00 ± 2.556	60.33 ± 1.02	27.31
Heritability	0.932	0.978	-	0.00	0.824	-
Mean	0.585	0.851	-	80.73	70.833	-
Range (Mean)	0.43–0.80	0.63–1.35	-	76.67–83.67	60.33–77.67	-
SE	0.024	0.915	-	1.970	35.8286	-
Range (SE)	0.003–0.075	0.004–0.17	-	0.840–2.556	0.88–4.09	-
LSD (0.05)	0.09 **	0.092 **	-	6.46 ^ns^	6.99 **	-
CV (%)	9.756182	6.317	-	4.66	5.75	-

Values are means ± SE. (*n* = 3), ** *p* ≤ 0.001; ns., not significant, HT is heat tolerant, HS is heat susceptible.

**Table 9:** Mean values of membrane thermostability index (MSI), and pollen viability with standard error and their reduction or increase in percent of 10 screened lentil genotypes (eight HT, and two HS) under OS and LS conditions during the 2020–2021 crop growing season at PRC, Ishurdi, Pabna.

Genotypes	Membrane Thermostability Index (MSI)			Pollen Viability (%)		
	OS	LS	Increasing (%)	OS	LS	Reduction
BLX 09015 (HT)	12.67 ± 0.12	18.3 ± 0.22	44.44	77.29 ± 0.826	74.71 ± 0.31	3.34
PRECOZ (HT)	13.93 ± 0.09	20.23 ± 0.18	45.23	82.24 ± 0.877	76.11 ± 0.26	7.45
BLX 05002-3 (HT)	12.37 ± 0.26	19.33 ± 0.21	56.27	82.51 ± 0.388	76.32 ± 0.41	7.50
LRL-21-112-1-1-1-1-6 (HT)	12 ± 0.25	17.77 ± 2.91	48.08	86.48 ± 0.696	77.44 ± 11.77	10.45
LR-9-25 (HT)	13.7 ± 0.10	20.23 ± 0.18	47.66	72.04 ± 0.899	66.65 ± 0.21	4.71
BLX 05002-6 (HT)	12.6 ± 0.17	18.1 ± 0.15	43.65	83.63 ± 1.056	69.96 ± 0.46	4.39
BARI Masur-8 (HT)	14.73 ± 0.23	20.9 ± 0.49	41.89	86.62 ± 0.524	71.17 ± 0.81	17.84
RL-12-181 (HT)	15.53 ± 0.18	23 ± 0.50	48.10	84.73 ± 0.557	66.68 ± 0.41	17.74
BLX 12009-6 (HS)	13.93 ± 0.33	25.67 ± 0.35	84.28	85.84 ± 0.982	68.86 ± 0.41	19.78
LG-198 (HS)	14.17 ± 0.30	27.73 ± 0.32	95.70	81.06 ± 1.276	66.43 ± 0.09	21.60
Heritability	0.96225	0.978	-	0.963757297	0.973	-
Mean	13.56333	21.167	-	82.243	71.433	-
Range (Mean)	12.00–15.53	18.10–27.73	-	72.04–86.62	66.43–77.44	-
SE	0.203	11.0522	-	0.88	36.2029	-
Range (SE)	0.09–0.33	0.26–5.01	-	0.388–1.276	0.283–1.39	-
LSD (0.05)	0.65 **	1.489 **	-	2.59 **	2.15 **	-
CV (%)	2.80641	4.11	-	1.83	1.76	-

Values are mean ± SE. (*n* = 3), ** *p* ≤ 0.001; HT is heat tolerant, HS is heat susceptible.

## Data Availability

Data will be available after request.

## Supplementary Materials

The following supporting information can be downloaded at: https://www.mdpi.com/article/10.3390/life12111719/s1, Table S1: List of 60 lentil genotypes with the source of collection, and present status; Table S2: List of screened genotypes as tolerant and susceptible to terminal heat stress used for physiochemical and reproductive trait study during 2020–21 at PRC, BARI, Ishurdi, Pabna; Table S3: The monthly average temperature at the control and polythene shades with relative humidity, sunshine hours and total rainfall (at the control) during crop season 2020–2021 at PRC, BARI, Ishurdi, Pabna
