# Supplementary material for: Selection of Lentil (Lens Culinaris (Medik.)) Genotypes Suitable for High-Temperature Conditions Based on Stress Tolerance Indices and Principal Component Analysis
Source: Life (Basel). 2022 Oct 27;12(11):1719. doi: 10.3390/life12111719 (PMC9698439; doi:10.3390/life12111719)
Supplement: Supplementary file 1 [file life-12-01719-s001.zip › life-1909020-supplementary.pdf]

Table SI: List of using 60 lentil genotypes with source of collection, and present status

Sl. No.	Accession code or name	Source/Origin	Status
1	BARI Masur-3	PRC	Popular variety
2	BARI Masur-4	PRC	Variety
3	BARI Masur-5	PRC	Variety
4	BARI Masur-6	PRC	Popular variety
5	BARI Masur-7	PRC	Popular variety
6	BARI Masur-8	PRC	Popular variety
7	BARI Masur-9	PRC	Short duration variety
8	LRIL-21-67-1-1-1-1	ICARDA	
9	LRIL-21-112-1-1-1-1-6	ICARDA	
10	IRLL-22-68-1-1-1-1-0	ICARDA	
11	ILL-8008	ICARDA	
12	ILL-5143	ICARDA	
13	91517	ICARDA	
14	9945	ICARDA	
15	LR-9-25	ICARDA	
16	PRECOZ	ICARDA	Popular variety in Argentina
17	Flip-2009-52L	ICARDA	
18	BLX-98001-9	PRC breeding material	
19	BLX-99033-19	PRC breeding material	
20	BLX-01013-21-5	PRC breeding material	
21	BLX-01014-9	PRC breeding material	
22	BLX-02009-11-1	PRC breeding material	
23	BLX-02009-17-1	PRC breeding material	
24	BLX-0200-08-4	PRC breeding material	
25	BLX-0200-18-3	PRC breeding material	
26	BLX-04004-10	PRC breeding material	
27	BLX-04010-15	PRC breeding material	
28	BLX-05001-10	PRC breeding material	
29	BLX-05002-6	PRC breeding material	
30	BLX-05004-8	PRC breeding material	
31	BLX-05005-3	PRC breeding material	
32	BLX-05006-7	PRC breeding material	
33	BLX-05007-5	PRC breeding material	
34	BLX-05008-3	PRC breeding material	
35	BLX-05008-11	PRC breeding material	
36	BLX-05010-3	PRC breeding material	
37	BLX-05011-3	PRC breeding material	
38	BLX-05010-6	PRC breeding material	
39	BLX 01013	PRC breeding material	
40	BLX-12004-5	PRC breeding material	
41	BLX-12009-6	PRC breeding material	
42	BLX-12011-6	PRC breeding material	
43	L 4717	ICARDA	
44	Bagura Local	Bangladesh	Land race
45	RL-12-178	ICARDA	
46	RL-12-171	ICARDA	

Sl. No.	Accession code or name	Source/Origin	Status
47	Maitree	India	Popular variety in West Bengal, India
48	RL-12-181	ICARDA	
49	BLX 11014-8	PRC breeding material	
50	BLX 11014-10	PRC breeding material	
51	BLX 11014-11	PRC breeding material	
52	BLX 10001-1	PRC breeding material	
53	BLX 10002-15	PRC breeding material	
54	BLX 10002-20	PRC breeding material	
55	LRIL-18-102	ICARDA	
56	LRIL-22-158	ICARDA	
57	LG-198	ICARDA	
58	BLX-05002-3	PRC breeding material	
59	BLX 09015	PRC breeding material	
60	BLX 04005-9	PRC breeding material	

Table S2: List of screened genotypes as tolerance and susceptible to the terminal heat stress used for physiochemical and reproductive traits study during 2020-21 at PRC, BARI, Ishurdi, Pabna

Sl. No.	Genotypes name	Heat stress reaction
01.	BLX 09015	Tolerance
02.	PRECOZ	Highly tolerance
03.	BLX-05002-3	Highly tolerance
04.	LRL-21-112-1-1-1-1-6	Highly tolerance
05.	LR-9-25	Highly tolerance
06.	BLX 05002-6	Highly tolerance
07.	BARI Masur-8	Highly tolerance
08.	RL-12-181	Highly tolerance
09.	BLX 12009-6	Susceptible
10.	LG-198	Susceptible

Table S3. Monthly average temperature at control and polythene shade with relative humidity, sunshine hours and total rainfall (at control) during crop season 2020-2021 at PRC, BARI, Ishurdi, Pabna

Month	Temperature (^{0}C)				Relative humidity	Sunshine hours	Total rainfall
	Maximum		Minimum				
	Control	Polythene shade	Control	Polythene shade			
December 2020	25	28.6	14.2	17.5	89.5	6.1	0
January 2021	23.8	27	11.4	15.7	87.7	5	0
February 2021	28.7	31.9	13	17.5	79.13	7.8	0
March 2201	34.4	38.6	19.8	24.5	72.39	7.1	0
